# Radiation-induced upregulation of FGL1 promotes esophageal squamous cell carcinoma metastasis via IMPDH1

**DOI:** 10.1186/s12885-024-12313-7

**Published:** 2024-05-03

**Authors:** Shan Huang, Jiayi Zhang, Pu He, Xinyue Cui, Yuzhu Hou, Wanghui Su, Fang Li

**Affiliations:** 1 Department of Radiation Oncology, Second Affiliated Hospital, Xi’an Jiaotong University, No.157, Xi Wu Road, Xi’an, 710004 ShaanXi China; 2 Department of Pathogenic Microbiology and Immunology, School of Basic Medical Sciences, Xi’an Jiaotong University, Xi’an, ShaanXi China

**Keywords:** Esophageal squamous cell carcinoma, FGL1, Radiation therapy, IMPDH1, Metastasis

## Abstract

**Background:**

While radiation therapy remains pivotal in esophageal squamous cell carcinoma (ESCC) treatment, the perplexing phenomenon of post-radiation metastasis presents a formidable clinical challenge. This study investigates the role of fibrinogen-like protein 1 (FGL1) in driving ESCC metastasis following radiation exposure.

**Methods:**

FGL1 expression in post-radiation ESCC cells was meticulously examined using qRT-PCR, western blotting, and immunofluorescence. The impact of FGL1 on ESCC cell invasion and migration was assessed through Transwell and wound healing assays. In vivo, the metastatic potential of ESCC in response to FGL1 was scrutinized using nude mice models. Comprehensive RNA sequencing and functional experiments elucidated the intricate mechanism associated with FGL1.

**Results:**

Radiation induced upregulation of FGL1 in ESCC cells through FOXO4, intensifying ESCC cell invasion and migration. Targeted knockdown of FGL1 effectively alleviated these characteristics both in vitro and in vivo. FGL1 depletion concurrently suppressed IMPDH1 expression. Rescue experiments underscored that IMPDH1 knockdown robustly reversed the pro-invasive effects induced by FGL1 in ESCC cells. ESCC tissues exhibited heightened IMPDH1 mRNA levels, demonstrating a correlation with patient survival.

**Conclusions:**

Radiation-induced upregulation of FGL1 propels ESCC metastasis through IMPDH1, proposing a potential therapeutic target to mitigate post-radiotherapy metastasis in ESCC patients.

**Supplementary Information:**

The online version contains supplementary material available at 10.1186/s12885-024-12313-7.

## Introduction

Esophageal cancer ranks as the seventh most prevalent cancer globally [1], with esophageal squamous cell carcinoma (ESCC) exhibiting a higher incidence in Asian populations and associated with relatively higher mortality rates [2]. Despite extensive research efforts, therapeutic outcomes for ESCC have shown limited improvement over recent decades compared to other extensively studied cancer types [3]. Recent studies have highlighted the significant roles of molecular mechanisms and genetic factors in the pathogenesis, progression, and treatment of ESCC. Analysis of 139 ESCC cases revealed frequent dysregulation of multiple molecular pathways, including the RTK-MAPK-PI3K pathway, cell cycle regulation, and epigenetic modifications [4]. Understanding the genetic abnormalities and molecular basis of ESCC is crucial for elucidating its pathophysiological processes and designing more effective targeted therapies. Radiation therapy serves as a cornerstone in the treatment of ESCC, traditionally focusing on inducing direct damage and cell death in tumor cells [5, 6]. However, emerging research suggests that radiation therapy may exert broader effects on tumor biology, potentially influencing tumor invasion and metastasis [7–10]. This intriguing possibility has prompted extensive exploration into the molecular mechanisms involved, with particular emphasis on the roles of fibrinogen-like protein 1 (FGL1) and inosine monophosphate dehydrogenase 1 (IMPDH1).

Fibrinogen like 1 (FGL1), a member of the fibrinogen family [11], exerts crucial regulatory roles in cell mitosis and metabolism [12, 13]. Recent studies have unveiled elevated FGL1 expression in various malignancies [14, 15], with heightened levels correlated with increased tumor invasion and metastasis [16]. Additionally, correlations have been established between FGL1 expression levels and clinical stage as well as patient outcomes in gastric cancer [16] and hepatocellular carcinoma [17]. Notably, studies have observed a notable upregulation of FGL1 expression in response to radiation, particularly evident across diverse biological contexts [18–20]. Despite the extensive exploration of FGL1 in various scenarios, its behavior in the context of radiation therapy, including its intricate interplay with molecules like IMPDH1, remains further investigation.

IMPDH1 is a critical component of the purine nucleotide synthesis pathway [21]. It plays a vital role in the de novo production of purine nucleotides, which are essential for processes like DNA and RNA synthesis and energy metabolism [22–24]. Notably, during periods of rapid cell proliferation, such as immune responses to antigens, there is an escalated demand for purine nucleotides [25–27]. It has been observed that IMPDH1 forms cytoplasmic structures during these processes, thereby facilitating the increased consumption of purine nucleotides [28]. Furthermore, the upregulation of IMPDH1 has been linked to uncontrolled cell proliferation, suggesting its potential importance in tumor initiation and progression [29, 30]. However, a thorough investigation is necessary to elucidate the specific roles and underlying mechanisms of IMPDH1 in the context of cancer.

This study unveils the significant upregulation of FGL1 after exposure to radiation and elucidates its impact on ESCC cell migration and metastasis, using both in vitro and in vivo. Additionally, our study elucidated FGL1’s role in regulating IMPDH1 expression, potentially identifying FGL1-IMPDH1 as a promising therapeutic target for ESCC.

## Materials and methods

### Cell culture and X-ray radiation treatment

ESCC cell lines (TE1 and ECA109) were procured from the National Collection of Authenticated Cell Cultures and maintained in our laboratory. These cell lines were cultured in a controlled environment at 37 °C with 5% CO_2_. TE1 cells were cultured in RPMI-1640 medium with 10% fetal bovine serum (FBS), while ECA109 cells were cultured in DMEM/high-glucose medium. ESCC cells were received 6MV X-ray radiation at a dose of 6–8 Gy, using a linear accelerator (Elekta Instruments, Inc., Stockholm, Sweden).

### Lentiviral transfection for FGL1 expression modulation in ESCC cells

Lentiviral vectors were meticulously designed and constructed for both FGL1 overexpression (FGL1-OE) and knockdown (FGL1-KD) using a lentivirus system obtained from Hanbio Biotechnology Co., Ltd. (Shanghai, China). Plasmids carrying the target genes for FGL1 overexpression and the shRNA constructs for FGL1 knockdown were carefully prepared. Plasmids carrying the target genes for FGL1 overexpression and the shRNA constructs for FGL1 knockdown were carefully prepared. Plasmids carrying random nonsense sequences served as non-specific control (NC). These constructs were then transfected into HEK293T cells, chosen for their high transfection efficiency in lentivirus production. Over a 48-hour incubation period, HEK293T cells produced lentivirus particles containing the desired genetic alterations. The lentiviral supernatants were collected from the cell culture medium and subsequently used to infect ESCC cells. Stable ESCC cell lines with modified FGL1 expression were established through puromycin selection. The success of the transfection and its resulting impact on FGL1 expression were verified through molecular techniques, including PCR and western blotting analysis. This comprehensive approach ensured the successful establishment of an ESCC cellular model with controlled FGL1 expression levels, which serves as a crucial foundation for subsequent experimental investigations.

### RNA isolation and quantitative real-time PCR (qRT-PCR)

The cDNA was synthesized from isolated RNA samples using the PrimeScript™ RT Reagent Kit (Takara Bio Inc., Shiga, Japan). qRT-PCR was performed using SYBR Premix Ex Taq™ II (Takara Bio Inc., Shiga, Japan), with β-actin used as the internal reference for normalization. Detailed primer sequences are provided in Table S1.

### shRNA transfection

For IMPDH1 or Forkhead box O4 (FOXO4) knockdown, shRNAs targeting IMPDH1( sh-IMPDH1), FOXO4 (sh-FOXO4) and non-specific control (sh-NC) were sourced from Genechem Co., Ltd.(Shanghai, China). Transfection was conducted employing Lipofectamine™3000 (Invitrogen, Thermo Fisher Scientific, Waltham, MA, USA) following established protocols. Briefly, selected shRNA sequences were complexed with Lipofectamine™3000 and added to the cells, facilitating shRNA uptake. Post-transfection for 72 h, the culture medium was refreshed to remove the transfection reagent. Detailed shRNA sequences are provided in Table S2.

### Protein analysis by western blotting

Our western blotting experiments adhered to established protocols [31]. In brief, we extracted proteins from ESCC cells using RIPA lysis buffer (Thermo Fisher Scientific, Waltham, MA, USA) and subsequently subjected them to electrophoresis on a 15% SDS-PAGE gel. These proteins were then transferred onto a PVDF membrane. Prior to antibody hybridization, the membranes were cut to focus on the target bands. Following blocking, we incubated the membranes with primary antibodies, including anti-FGL1 (1:1000, ab197357, Abcam, Cambridge, United Kingdom), anti-FOXO4 (1:1000, 21535-1-AP, Proteintech, Rosemont, IL, USA) and anti-IMPDH1 (1:1000, 22092-1-AP, Proteintech, Rosemont, IL, USA), for 14 h. Subsequently, we employed secondary antibodies for a 1-hour incubation at room temperature. Signal detection was conducted using the ECL kit (Millipore, Massachusetts, USA). Data analysis was performed using NIH-ImageJ software.

### Site-directed mutagenesis and reporter gene assay

Site-directed mutations of the FOXO4- binding site within the FGL1 promoter region was performed by Hanbio Biotechnology (Hanbio Biotechnology Co., Ltd., Shanghai, China). The gene sequences of h-FGL1-pro-WT and h-FGL1-pro-MUT are in Appendix file 2. We constructed the firefly Luciferase (Fluc) reporter vector of h-FGL1-pro-WT and h-FGL1-pro-MUT as well as pcDNA3.1-FOXO4 overexpression vector. The constructed vectors were confirmed with DNA sequencing. Transcriptional activity was evaluated through the application of a luciferase assay system. The h-FGL1-pro-WT or MUT vector and pcDNA3.1-FOXO4 were co-transfected into 293T cells, with ranilla luciferase (Rluc) RL-TK used as an internal control. After a 48-hour incubation period following transfection, the quantification of fluorescence intensities was executed using the Dual-Luciferase® Reporter Assay System (Hanbio Biotechnology Co., Ltd., Shanghai, China).

### Cellular immunofluorescence staining

ESCC cells were cultured in laser confocal-specific cell culture dishes (801,001, NEST Life Science Technology Co., Ltd., Wuxi, Jiangsu, China) until they adhered to the surface. Subsequently, they were washed with phosphate-buffered saline (PBS) and air-dried. Fixation was carried out using 4% paraformaldehyde for 20 min. Following fixation, permeabilization was achieved by employing a 0.1% Triton X-100 solution. Following a thorough washing step, the cells underwent a 30-minute blocking procedure at room temperature using a 5% bovine serum albumin solution. Subsequently, the cells were incubated with primary anti-FGL1 antibodies (1:1000, ab197357, Abcam, Cambridge, UK) at 4 °C overnight. Cells were incubated with secondary antibodies conjugated with fluorescent markers (1:2000, ab150077, Abcam, Cambridge, United Kingdom) at room temperature for 1 h. DAPI (C0065-10ML, Beijing Solarbio Science & Technology Co., Ltd, Beijing, China) was used to visualize the cell nuclei for 10 min. Visualization and imaging were performed using a laser confocal fluorescence microscopy (NIKON A1, Nikon, Tokyo, Japan).

### Transwell invasion assay

A Transwell invasion assay was employed to evaluate cell invasiveness. Prior to cell inoculation, 50 µL of Matrigel (BD Biosciences, San Jose, California, USA) was applied to the upper membrane of the Transwell chamber. The cells designated for testing were prepared as a single-cell suspension without serum. A total of 2 × 10^5^ cells were seeded into the upper compartment of the Transwell unit (8-micron pore size, Corning, New York, USA), and a medium containing 10% FBS was added to the lower chamber. The inoculated Transwell chambers were then incubated in a cell culture incubator. After 24 h, the Transwell chambers were carefully removed, and cells on the upper side of the membrane were gently swabbed. Cells that had migrated through the Transwell membrane were fixed with a 4% paraformaldehyde solution and stained with 0.05% crystal violet. Subsequently, they were observed under a microscope.

### Wound healing assays

A wound healing assay was conducted to evaluate cell migration capabilities. The cells to be tested were cultured in a 6-well plate until they reached approximately 80% confluence. Once the cells had adhered to the surface and reached this confluence, a sterile pipette tip was used to create uniform scratches in the monolayer. The detached cells were removed using PBS, and serum-free medium was added to sustain further growth. The extent of cell migration into the scratched area was observed at specific time points, and the width of the scratch was measured.

### RNA sequencing

Total RNA samples were extracted, subjected to agarose gel electrophoresis, and quantified using Nanodrop. mRNA enrichment was performed using oligo(dT) magnetic beads. RNA sequencing libraries were prepared using a kit, including RNA fragmentation, first-strand cDNA synthesis using random primers, second-strand cDNA synthesis with dUTP incorporation, end repair, A-tailing, and adapter ligation for Illumina sequencing. The final library was generated through PCR amplification. Quality control of the constructed libraries was conducted using the Agilent 2100 Bioanalyzer, followed by library quantification using qPCR methods. Sequencing of the prepared libraries was performed using the Illumina NovaSeq 6000 sequencer. The sequenced data were submitted to the GEO database for further analysis (GSE264221).

### Animal lung metastasis assessment

Four-week-old male BALB/c nude mice were procured from the Animal Experiment Center at the School of Medicine, Xi’an Jiaotong University, China. The objective was to evaluate how the absence of FGL1 influences the metastatic potential of ESCC cells. To achieve this, the mice received tail vein injections of 5 × 10^7^ ECA109 cells. The experimental group received FGL1-KD ECA109 cells, while the control group received NC ECA109 cells. After the manifestation of lung metastasis in the mice, humane euthanasia was performed, followed by surgical removal of lung tissues, and subsequent calculation of metastatic nodules. The euthanasia procedure adhered to ethical guidelines and utilized the carbon dioxide (CO_2_) inhalation method, as recommended by the AVMA Guidelines for the Euthanasia of Animals: 2020 Edition. Animals were placed in a chamber pre-filled with CO_2_ gas at a concentration of 30% of the chamber volume per minute, ensuring rapid and painless euthanasia while minimizing animal suffering. All experimental protocols were conducted in strict accordance with the ethical guidelines and regulations approved by the Medical Ethics Committee of Xi’an Jiaotong University.

### Statistical analysis

Statistical analysis was conducted utilizing GraphPad Prism 9.5.1 software. Data distribution normality was assessed using the Shapiro-Wilk test. Results are presented as mean ± standard error of the mean (SEM) from at least three independent experiments. Statistical distinctions were determined using Student’s t-test or one-way analysis of variance (ANOVA) for data that adhered to normal distribution. In instances where the data deviated from a normal distribution, the non-parametric Mann-Whitney U test was employed, and outcomes were reported as medians. We employed Logrank tests to evaluate the significance of Kaplan-Meier survival curves. All statistical tests were two-tailed, and a p-value below 0.05 was deemed statistically significant.

## Results

### Radiation-induced upregulation of FGL1 in ESCC cells and its regulation by FOXO4

**Fig. 1:**
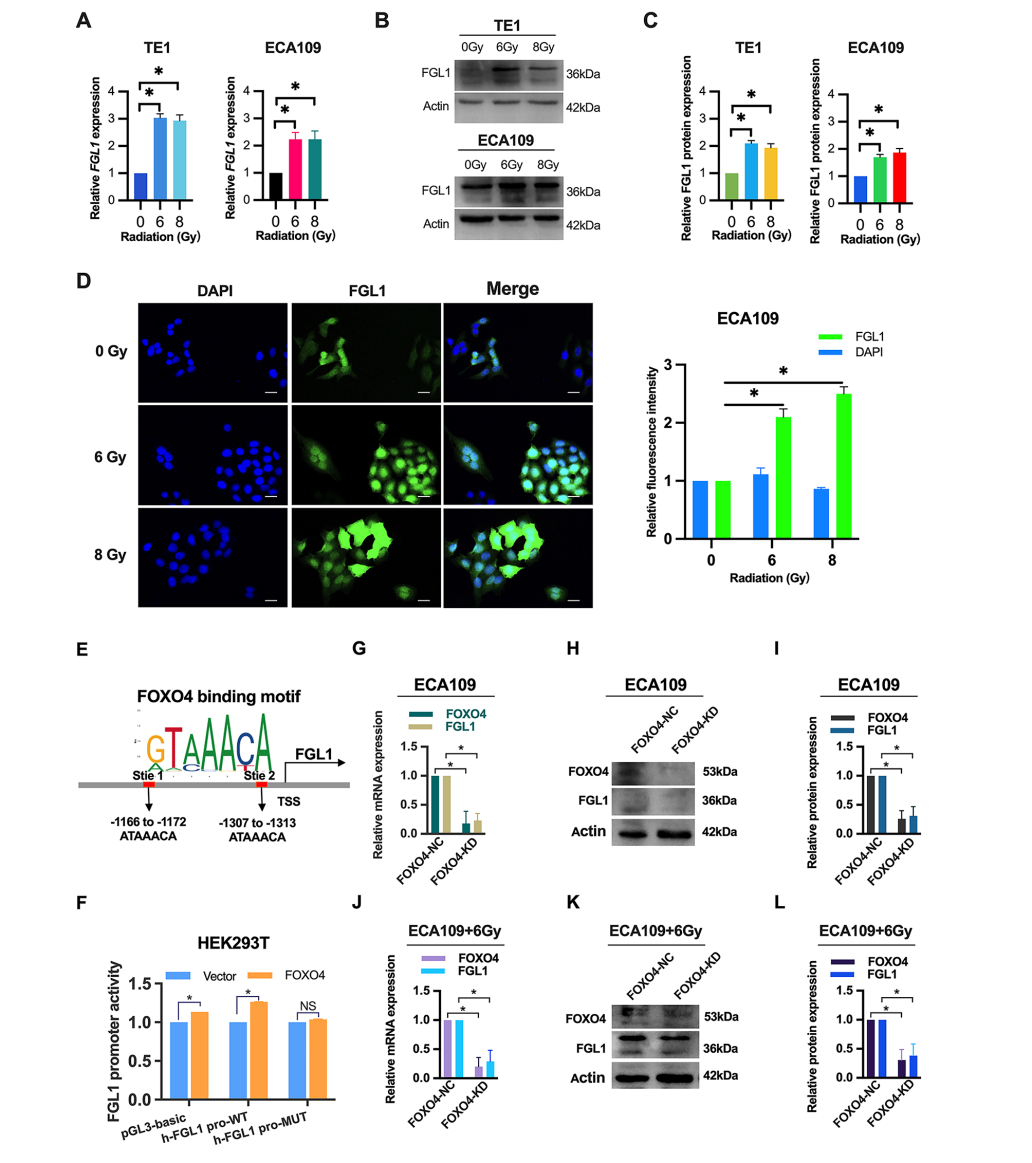
Radiation enhances FGL1 expression in ESCC cells through FOXO4. (**A**) The expression level of FGL1 mRNA was detected by qRT-PCR at 48 h after X-ray radiation. (**B-C**) The expression level of FGL1 protein was detected by western blotting at 48 h after X-ray radiation. (**D**) The FGL1 protein expression in ECA109 cells by immunofluorescence staining at 48 h after X-ray radiation. Scale bars, 25 μm. (**E**) Schematic illustration of the predicted binding site of FOXO4 in the FGL1 promoter region by JASPAR analysis. (**F**) Luciferase reporter assay demonstrating the impact of FOXO4 motif mutations on FGL1 promoter activity in KET293T cells. (**G-I**) The expression level of FGL1 and FOXO4 were assessed in ECA109 cells transfected with FOXO4 shRNA (FOXO4-KD) or random nonsense sequences (FOXO4-NC) using qRT-PCR (**G**) and western blotting (**H** and **I**). (**J-L**) The expression level of FGL1 and FOXO4 at 48 h after 6 Gy X-ray radiation were assessed in ECA109 cells transfected with FOXO4 shRNA (FOXO4-KD) or random nonsense sequences (FOXO4-NC) using qRT-PCR (**J**) and western blotting (**K** and **L**). The statistical difference was assessed with one-way ANOVA followed by Dunnett tests in **A**, **C** and **D**; two-tailed unpaired Student t test in **F**, **G**, **I**, **J** and **L**. Error bars show the SD from three independent experiments. *p < 0.05

Given the central role of radiotherapy as a primary adjuvant approach in ESCC treatment, we examined the impact of X-ray radiation on FGL1 expression. PCR analysis showed a notable upregulation of FGL1 mRNA levels in ESCC TE1 and ECA109 cells following radiation (Fig. 1A). Western blotting analysis further illustrated a substantial increase in FGL1 protein expression post-radiation (Fig. 1B and C). Cellular immunofluorescence analysis confirmed a marked increase in FGL1 protein expression levels in ECA109 cells after radiation (Fig. 1D). Collectively, these findings establish that radiation induces upregulation of FGL1 expression.

To elucidate the molecular mechanisms underlying FGL1 upregulation, we conducted a thorough analysis of potential transcription factors using JASPAR programs(http://jaspar.genereg.net/). Our analysis identified a canonical binding motif for FOXO4 as the most promising candidate within the FGL1 promoter region (Table S3). Supporting FOXO4’s regulatory role, luciferase reporter assays demonstrated that FOXO4 overexpression significantly enhanced luciferase activity under the wild-type FGL1 promoter, while the mutated counterpart showed minimal changes (Fig. 1E and F). Loss-of-function experiments involving FOXO4 knockdown through shRNA-mediated silencing in ECA109 cells (Fig. 1G-I) revealed a substantial decrease in both FGL1 mRNA and protein levels both radiation (Fig. 1J-L), suggesting FOXO4’s direct involvement in radiation-induced FGL1 transcriptional activation. In summary, our study strongly supports radiation-induced upregulation of FGL1 in ESCC cells through FOXO4.

### FGL1 enhances ESCC cell invasion and migration

**Fig. 2:**
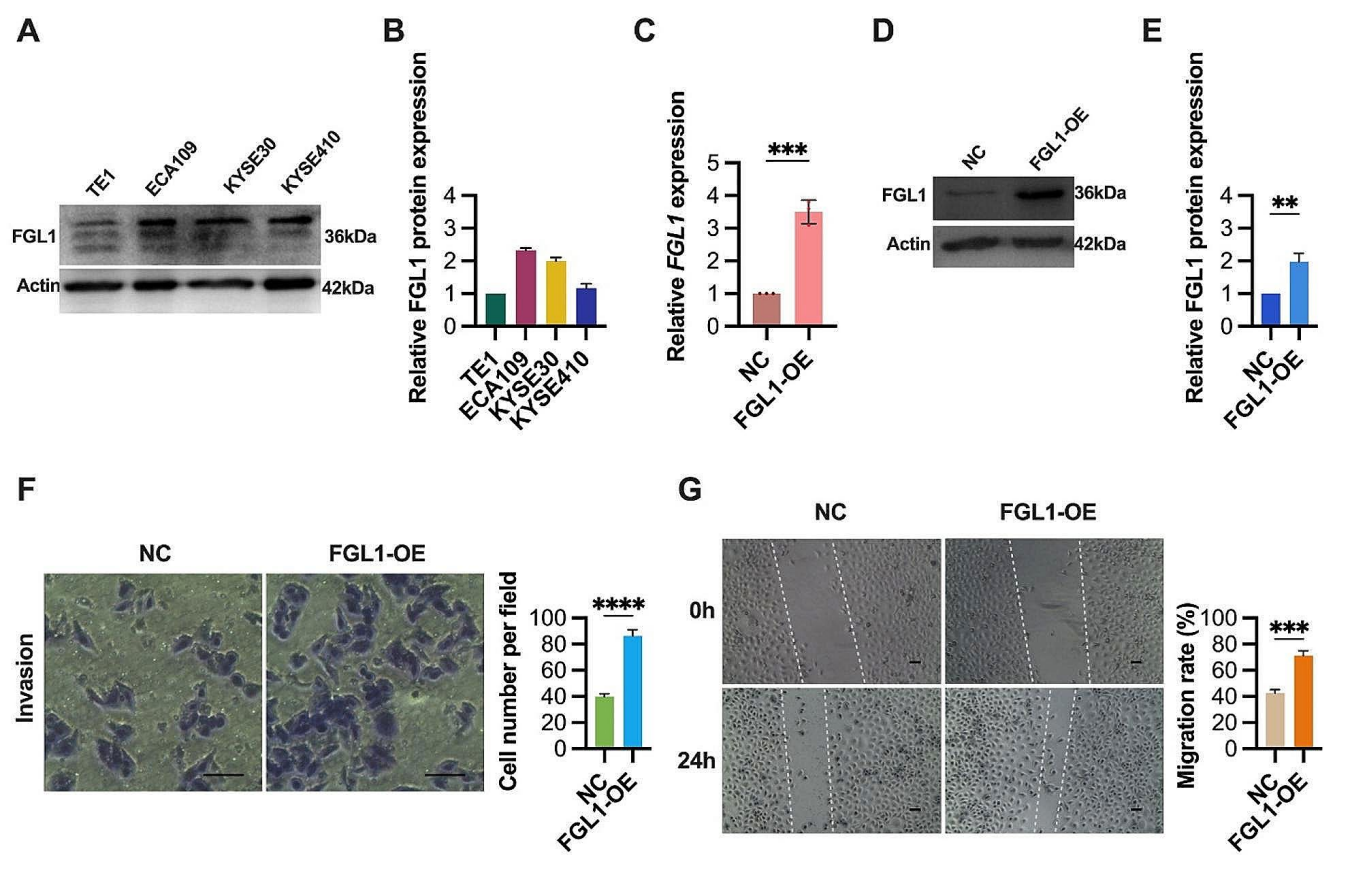
FGL1 promotes invasion and migration of ESCC cells. (**A-B**) FGL1 expression levels were assessed in ESCC cell lines by western blotting. β-Actin served as the loading control. (**C-E**) FGL1 expression levels were assessed in TE1 cells transfected with FGL1 (FGL1-OE) or random nonsense sequences (NC) using qRT-PCR (**C**) and western blotting (**D** and **E**). (**F**) The effects of FGL1 overexpression on TE1 cells invasion were evaluated by transwell invasion assay. Scale bars, 100 μm. (**G**) The effects of FGL1 overexpression on TE1 cells migration were evaluated by wound healing assay. Scale bars, 100 μm. The statistical difference was assessed with two-tailed unpaired Student t test in **C**, **E**, **F** and **G**. Error bars show the SD from three independent experiments. **p < 0.01, ***p < 0.001, ****p < 0.0001

In our investigation of FGL1’s role in ESCC cells, we first conducted analyses of FGL1 expression across various wild-type ESCC cell lines. Our findings revealed that FGL1 expression was lowest in the TE1 cell line and highest in the ECA109 cell line (Fig. 2A and B). Based on these results, we selected the ECA109 cell line for FGL1 gene knockdown experiments, while the TE1 cell line was chosen for FGL1 overexpression. FGL1 overexpression was achieved using a lentivirus-mediated approach. FGL1 overexpression (FGL1-OE) was validated through PCR (Fig. 2C) and western blotting (Fig. 2D and E) in TE1 cells. Functional experiments revealed a significant increase in ESCC cell invasion and migration upon FGL1 overexpression. Transwell invasion assays demonstrated a notable enhancement of cell invasion capability post-FGL1 overexpression (Fig. 2F). Similarly, wound healing assays indicated a significant increase in cell migration upon FGL1 overexpression (Fig. 2G). These results confirm that upregulation of FGL1 enhances the invasive and migratory abilities of ESCC cells.

### Suppression of ESCC cell migration in vitro and in vivo through FGL1 knockdown

**Fig. 3:**
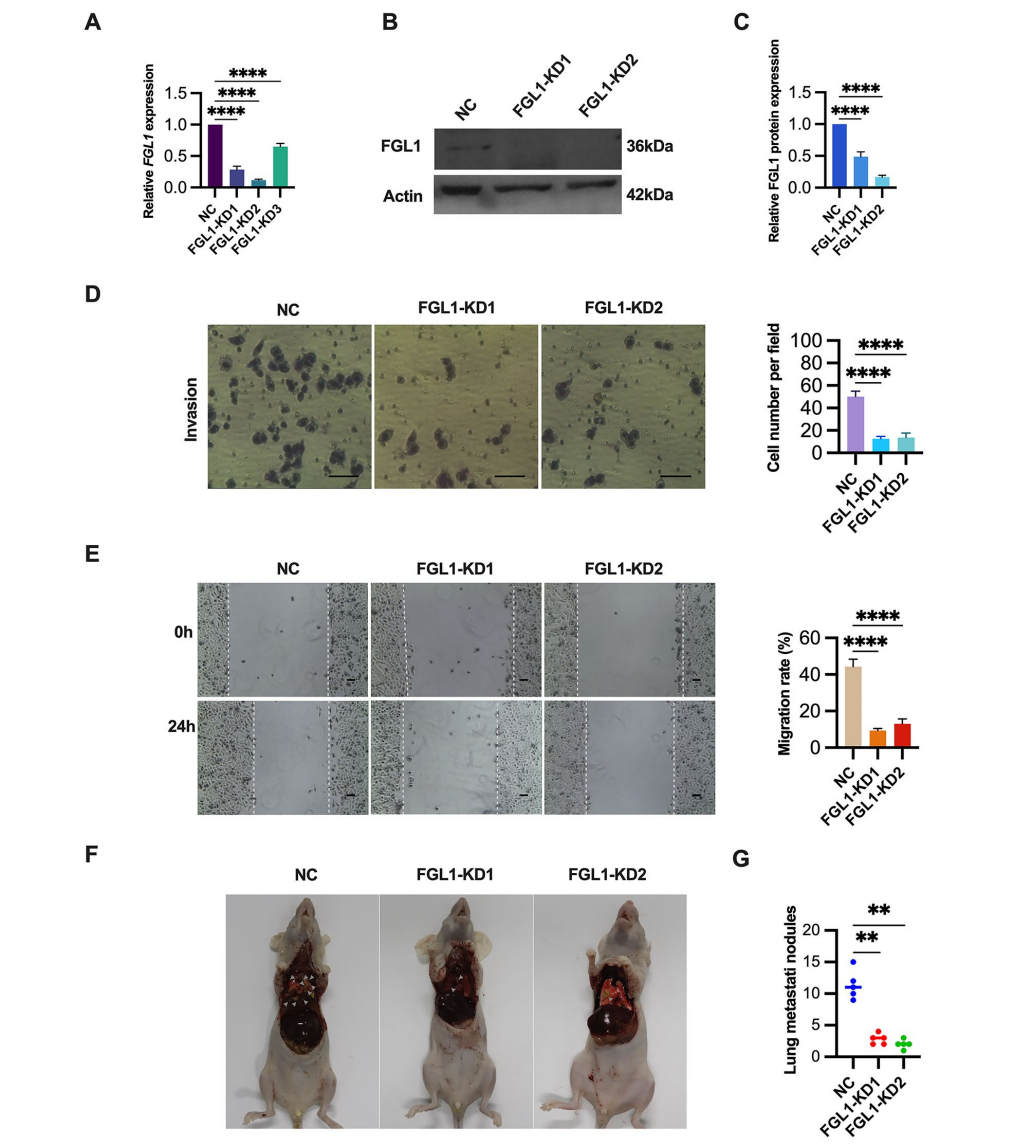
FGL1 knockdown inhibits invasion and migration of ESCC cells in vitro and in vivo. (**A-C**) FGL1 expression levels were assessed in ECA109 cells transfected with FGL1 shRNA (FGL1-KD) or random nonsense sequences (NC) using qRT-PCR (**A**) and western blotting (**B** and **C**). β-Actin served as the loading control. (**D**) The effects of FGL1 knockdown on ESCC cells invasion were evaluated by transwell invasion assay. Scale bars, 100 μm. (**E**) The effects of FGL1 knockdown on ESCC cells migration were evaluated by wound healing assay. Scale bars, 100 μm. (**F** and **G**) FGL1 knockdown resulted in decreased lung metastasis, shown by the representative image (**F**) and lung nodules number (**G**) of lung metastasis in nude mice bearing ECA109 cells. The statistical difference was assessed with one-way ANOVA followed by Dunnett tests in **A**, **C**, **D**, **E** and **G**. Error bars show the SD from three independent experiments. **p < 0.01, ****p < 0.0001

To gain deeper insights into the functions of FGL1 in ESCC, we employed lentivirus-mediated FGL1 gene silencing in ECA109 cells. Transfection efficiency was validated through PCR (Fig. 3A) and western blotting analyses (Fig. 3B and C). Subsequent functional assessments revealed a significant reduction in the migratory capabilities of ESCC cells in vitro following FGL1 knockdown. Transwell migration assays demonstrated a substantial decrease in cell migration ability after FGL1 knockdown (Fig. 3D). Similarly, wound healing migration assays indicated a significant decrease in cell migration following FGL1 knockdown (Fig. 3E). These findings underscore the inhibitory effect of FGL1 knockdown on ESCC cell migration in vitro and its role in suppressing metastasis in vivo, supported by a decrease in the count of lung metastatic nodules in the xenograft model (Fig. 3F and G).

### Radiation-induced regulation of IMPDH1 expression by FGL1

**Fig. 4:**
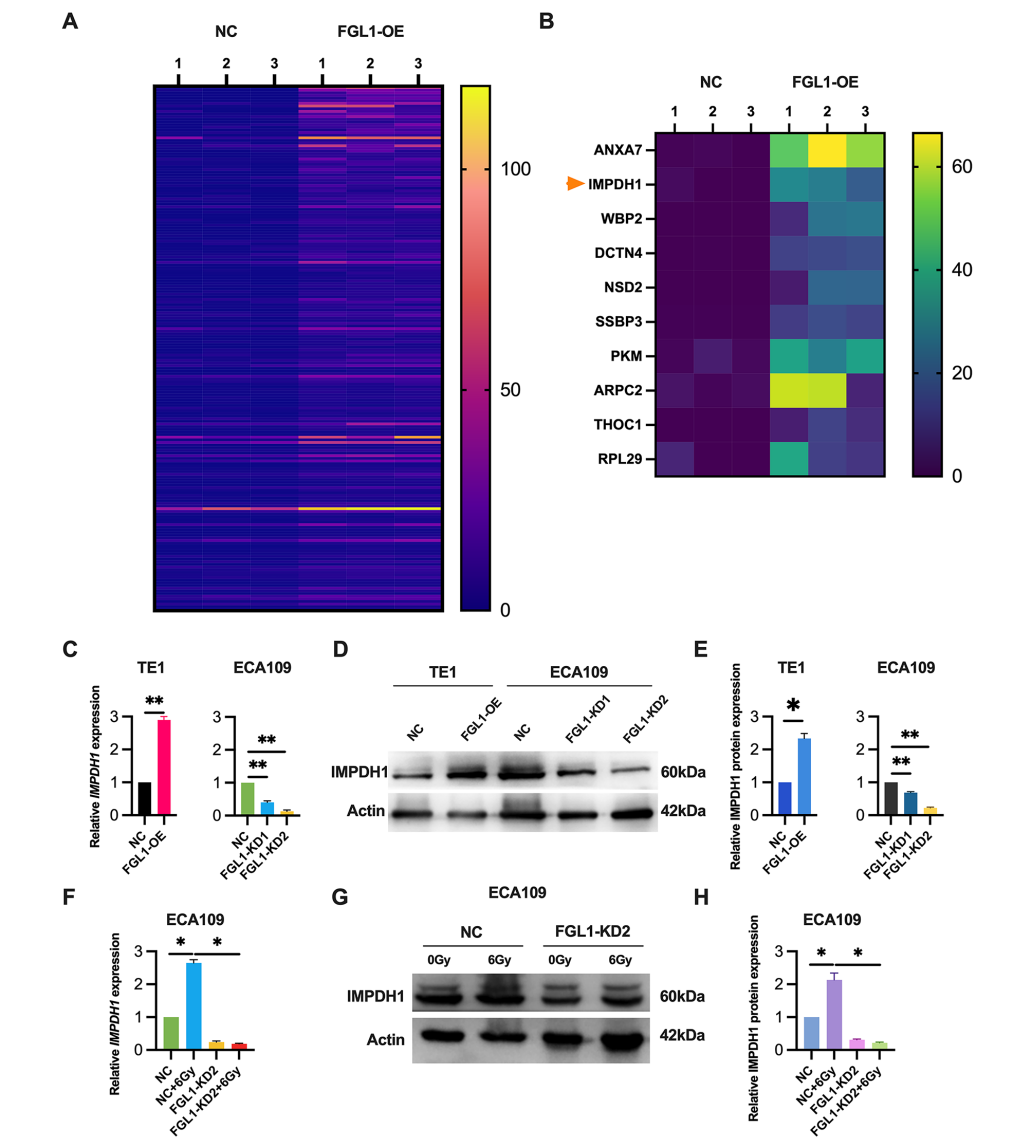
FGL1 modulates radiation-induced regulation of IMPDH1 expression. (**A**) Transcriptome analysis was performed using RNA-Seq to identify downstream target genes associated with FGL1 expression. The results unveiled a significant upregulation of 198 genes (FDR > 1, *p* < 0.05) following the overexpression of FGL1. (**B**) Top 10 upregulation target genes in (**A**) including IMPDH1. (**C-E**) IMPDH1 expression levels were assessed in TE1 cells transfected with FGL1 (FGL1-OE), and in ECA109 cells transfected with FGL1 shRNA (FGL1-KD) using qRT-PCR (**C**) and western blotting (**D** and **E**). β-Actin served as the loading control. (**F-H**) IMPDH1 expression levels were assessed in ECA109 cells transfected with FGL1 shRNA (FGL1-KD2) using qRT-PCR (**F**) and western blotting (**G** and **H**). β-Actin served as the loading control The statistical difference was assessed with the two-tailed unpaired Student t test in in C (left), **E** (left), **F** and **H**; and one-way ANOVA followed by Dunnett tests in **C** (right) and **E** (right). Error bars show the SD from three independent experiments. **p < 0.01

To unravel the mechanism by which FGL1 promotes cell migration in ESCC cells, we conducted a thorough RNA-seq analysis(GSE264221). Specifically, we induced the overexpression of FGL1 in TE1 cells, as depicted in Fig. 4A. Our analysis pinpointed the ten most significantly upregulated genes subsequent to FGL1 overexpression (Fig. 4B), placing specific emphasis on IMPDH1 due to its well-established crucial role in tumor metastasis according to prior research.

Ensuring the robustness and reliability of our findings, we conducted meticulous validation experiments across multiple ESCC cell lines. Initially, in TE1 cells with heightened FGL1 expression, both PCR and western blotting analyses confirmed the upregulation of IMPDH1 expression, as illustrated in Fig. 4C-E. Subsequently, to further strengthen our results, we carried out experiments in ECA109 cells where FGL1 was silenced. Here, both PCR and western blotting analyses revealed a notable reduction in IMPDH1 expression levels (Fig. 4C-E). These comprehensive results suggest that FGL1 knockdown leads to a significant suppression of IMPDH1 expression. Additionally, we investigated the impact of radiation on IMPDH1 expression, demonstrating an upregulation of IMPDH1 expression post-radiation(Fig. 4F-H) in ECA109 cells. Subsequent FGL1 gene knockdown experiments showed that the radiation-induced increase in IMPDH1 expression was attenuated, as evidenced by PCR (Fig. 4F) and western blotting analyses (Fig. 4G and H). Taken together, our findings suggest that FGL1 modulates radiation-induced regulation of IMPDH1 expression.

### FGL1 drives tumor metastasis through IMPDH1

**Fig. 5:**
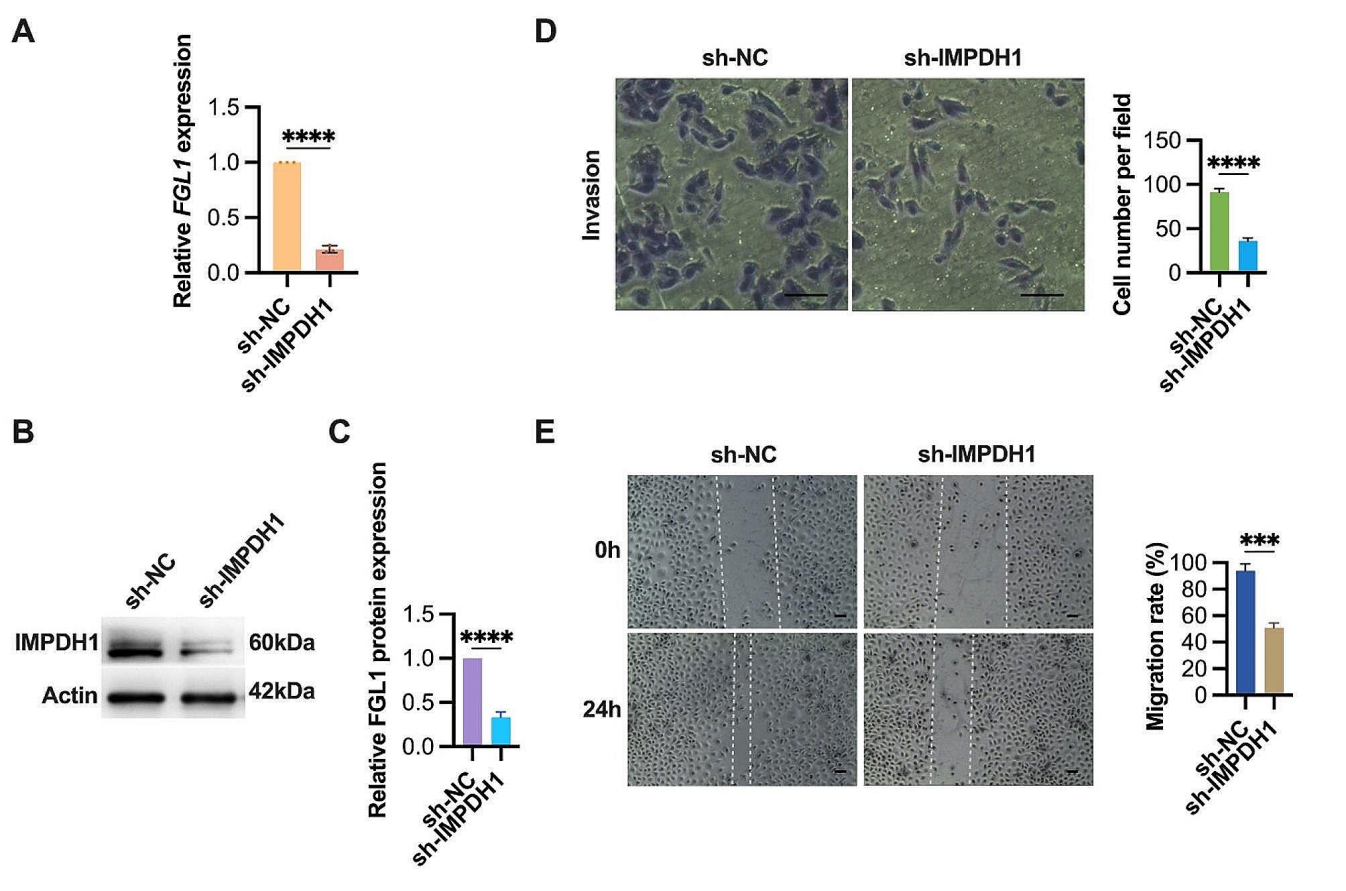
FGL1 drives invasion and migration through IMPDH1 in ESCC cells. (**A-C**) Quantification of IMPDH1 expression levels in FGL1-OE TE1 cells transfected with IMPDH1 shRNA (sh-IMPDH1) or random nonsense sequences (sh-NC) using qRT-PCR (**A**) and western blotting (**B** and **C**). β-Actin served as the loading control. (**D**) The effects of IMPDH1 knockdown on FGL1-OE TE1 cells invasion were evaluated by transwell invasion assay. Scale bars, 100 μm. (**E**) The effects of FGL1 knockdown on FGL1-OE TE1 cells migration were evaluated by wound healing assay. Scale bars, 100 μm. The statistical difference was assessed with the two-tailed unpaired Student t test in **A**, **C**, **D** and **E**. Error bars show the SD from three independent experiments. ***p < 0.001, ****p < 0.0001

In-depth exploration into the downstream mechanisms orchestrating FGL1’s impact on ESCC cell invasion and migration homed in on the role of IMPDH1. Utilizing shRNA-mediated knockdown, IMPDH1 was efficiently silenced in TE1 cells overexpressing FGL1, resulting in a substantial reduction in IMPDH1 mRNA expression (Fig. 5A) and protein levels (Fig. 5B and C), affirming the efficacy of this intervention.

Subsequent functional investigations unveiled a noteworthy attenuation in the pro-invasive effects of FGL1 with partial knockdown of IMPDH1. This attenuation was evident in a significant reduction in invasive potential, as assessed through transwell invasion assays (Fig. 5D). Furthermore, this intervention effectively countered the influence of FGL1 on cell migration, as confirmed by wound healing migration assays (Fig. 5E). These meticulous observations collectively underscore IMPDH1 as a critical downstream mechanism in the regulation of ESCC cell invasion and migration by FGL1. This provides valuable insights into the driving forces behind these phenomena and emphasizes IMPDH1’s pivotal role as a mediator of FGL1 in the regulation of ESCC cell invasion and migration processes.

### IMPDH1 expression and its potential prognostic implications in pan-cancer and ESCC

**Fig. 6:**
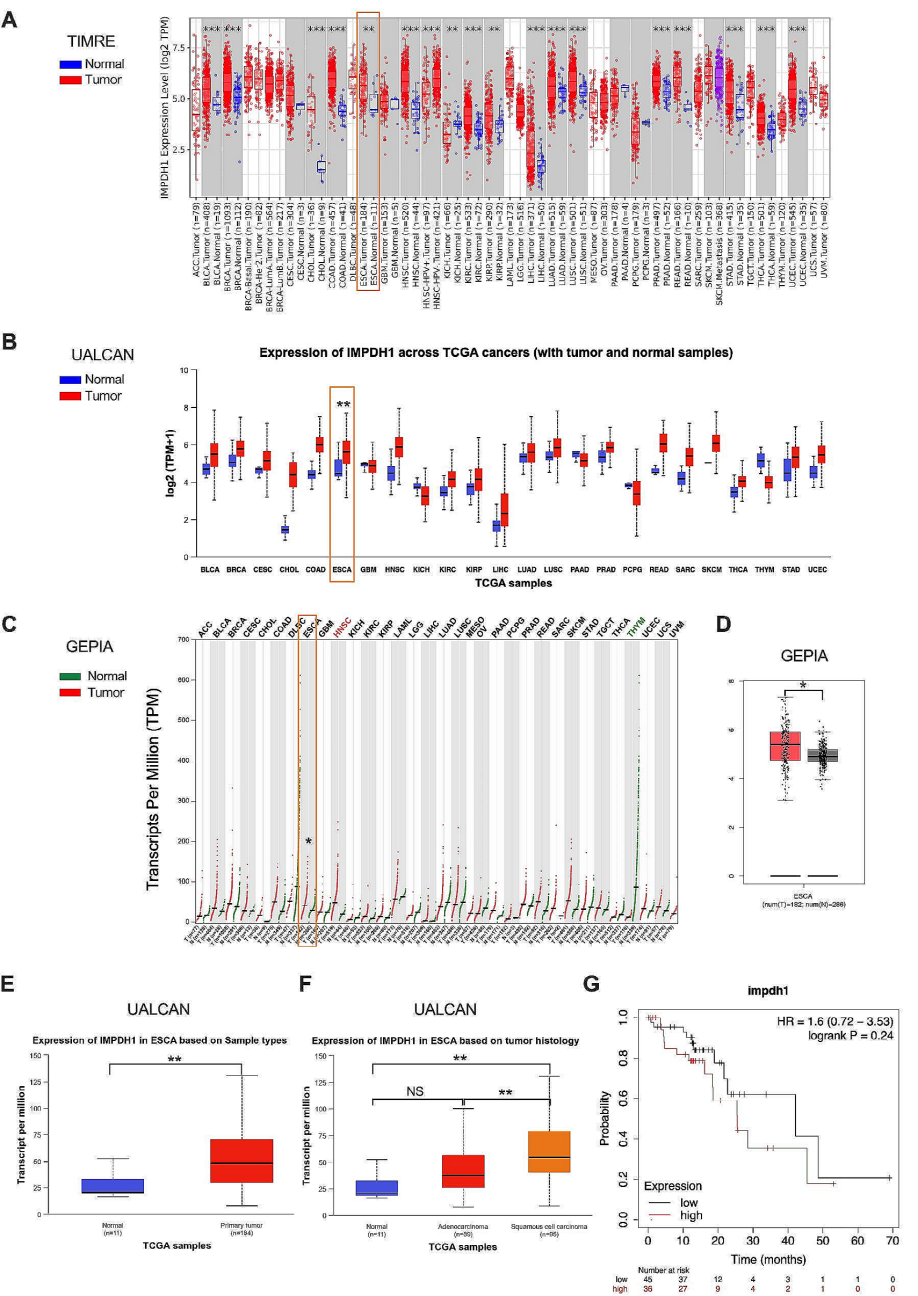
IMPDH1 is highly expressed in ESCC tissue and is related to the patient’s prognosis. (**A-C**) IMPDH1 expression in pan-cancer using TIMER2.0 **(A)**, UALCAN **(B)** and GEPIA **(C)** portals. (**D-E**) IMPDH1 expression in normal and esophageal carcinoma (ESCA) tissues using GEPIA **(D)** and UALCAN **(E)** portals. (**F**) IMPDH1 expression in esophageal squamous cell carcinoma (*n* = 95) and adenocarcinoma (*n* = 89) tissues using UALCAN portals. (**G**) Kaplan-Meier analyses of OS for esophageal carcinoma (ESCA) patients with high (*n* = 36) or low (*n* = 45) tumor IMPDH1 expression using TCGA database. The median survival time for low and high IMPDH1 expression cohort is 42.1 months and 25.5 months respectively. The statistical difference was assessed with the unpaired nonparametric Mann–Whitney U test in **A**, **B**, **C**, **D**, **E** and **F**; and the Logrank test for **G**. *p < 0.05, **p < 0.01; NS, not statistically significant

Building upon the established role of IMPDH1 in ESCC, we conducted a comprehensive analysis to assess its expression and potential prognostic implications. Employing various online tools, including TIMER2.0, UALCAN, and GEPIA, an extensive analysis of IMPDH1 gene mRNA expression across various cancer types was performed (Fig. 6A-C). The findings revealed elevated IMPDH1 expression in over two-thirds of pan-cancer cases compared to adjacent normal tissues.

Specifically in primary esophageal carcinoma tissues, IMPDH1 mRNA levels were significantly higher than those in adjacent normal tissues, underscoring its potential significance in esophageal carcinoma (Fig. 6D and E). Further analysis within the esophageal carcinoma subtype indicated notably higher IMPDH1 expression in squamous cell carcinoma compared to adenocarcinoma (Fig. 6F, *p* < 0.01). To deepen our understanding of the relationship between IMPDH1 and patient survival, Kaplan-Meier Plotter analysis was employed (https://kmplot.com/analysis). The analysis revealed a trend where patients with high IMPDH1 expression had less favorable overall survival (OS) compared to those with low IMPDH1 expression (Fig. 6G, HR = 1.6, 95% CI: 0.72–3.53). Notably, the lack of statistical significance may be attributed to the limited sample size (36 cases with high IMPDH1 expression and 45 cases with low IMPDH1 expression). In summary, these findings underscore the pivotal role of IMPDH1 in ESCC.

## Discussion

In this study, we aimed to investigate the intricate molecular mechanisms underlying the impact of radiation therapy on ESCC, with a specific focus on the role of FGL1 in tumor migration and metastasis. Our findings shed light on the direct association between FOXO4, FGL1 and IMPDH1, highlighting the potential implications of this relationship for the response to radiation therapy.

Our study unveils for the first time the radiation-induced upregulation of FGL1 expression in ESCC, a phenomenon predominantly observed in previous research in normal tissues such as the lungs, with limited exploration in the context of tumors [18–20]. Of particular note, we discovered the direct binding of FOXO4 to the promoter region of FGL1, enhancing its transcriptional activity. FOXO4, a key member of the FOXO transcription factor family [32], regulates vital biological processes including cell proliferation, apoptosis, and metabolism [33]. Despite its context-dependent roles in cancer development [34], FOXO4 involvement in cell cycle regulation and DNA repair renders it a potential therapeutic target [35, 36]. Our study highlights the specific interaction between FOXO4 and FGL1 under radiation therapy conditions, expanding our understanding of potential mechanisms underlying radiation response in the ESCC genome. In this context, FOXO4 emerges not only as a pivotal regulatory factor but also as a promising target for future therapeutic strategies.

Our functional experiments underscore the crucial role of FGL1 in promoting the migration and invasion of ESCC cells in vitro. These findings further elucidate FGL1’s involvement in ESCC progression, consistent with emerging evidence suggesting its role in facilitating tumor metastasis in other cancer types [15–17, 37]. Importantly, through in vitro and in vivo studies, we demonstrated that depletion of FGL1 inhibits ESCC cell metastasis, highlighting the potential of FGL1 depletion in enhancing patient survival and the development of novel therapeutic targets. Interestingly, our study revealed differential expression patterns of FGL1 in ESCC cells with distinct p53 statuses. TE−1 cells with low FGL1 expression harbor mutant p53, while ECA109 cells with high FGL1 expression carry wild-type p53. Previous research has shown that FGL1 induces the generation of regulatory T cells and promotes the formation of an immunosuppressive tumor microenvironment through interactions with various immune cells [14]. Recent studies have emphasized the critical role of p53 in tumor immune regulation [38]. This observation underscores the complex interplay between FGL1 and p53 in tumor biology and highlights the need for further investigation into their relationship. Upon re-analysis of RNA sequencing data (results not shown), we found an increase in KAI1, a p53 target gene involved in metastasis, in the group of ESCC cells with high FGL1 expression [39]. This suggests that FGL1 may modulate the tumor immune environment through complex regulatory mechanisms involving p53 in tumor immune regulation. Therefore, further research is warranted to explore the interaction between p53 and FGL1 and their impact on tumor immunity.

Our study reveals a mechanistic connection between FGL1 and IMPDH1, identifying IMPDH1 as a critical downstream regulator of FGL1. IMPDH1, a key enzyme in the nucleotide synthesis pathway, has been associated with cell proliferation and tumor development [24, 27, 30]. We demonstrate that radiation exposure induces the expression of both FGL1 and IMPDH1, while the radiation-induced increase in IMPDH1 expression is attenuated following FGL1 gene knockdown. Subsequent IMPDH1 knockdown experiments further support the relationship between these molecules, suggesting their involvement in radiation-induced metastasis. These findings are consistent with the concept proposed in existing literature targeting the IMPDH1/YB−1 axis to enhance metastatic renal cancer treatment [29]. From a clinical perspective, our results indicate the aberrantly high expression of IMPDH1 in esophageal cancer tissues and its close association with patient prognosis. By elucidating the molecular mechanisms underlying tumor progression and metastasis mediated by FGL1, our study contributes to a better understanding of the molecular pathways involved in post-radiation esophageal squamous cell carcinoma (ESCC), emphasizing the potential of FGL1/IMPDH1 as targets to mitigate radiation-induced tumor metastasis.

However, it’s important to note that our study carries certain limitations. We primarily focused on in vitro and in vivo experiments, and while these findings offer critical insights, further clinical investigations are paramount to confirm their clinical significance in human ESCC patients. Additionally, while we have identified IMPDH1 as a downstream regulator of FGL1, further elucidation of the exact molecular mechanisms governing their interaction remains necessary. Our research primarily concentrated on the impact of radiation-induced upregulation of FGL1, and future studies should investigate the effects of other therapeutic modalities on this pathway. Furthermore, although orthotopic and PDX models offer potential insights into tumor biology, their practical constraints led us to choose the tail vein injection model for studying metastasis in our specific context. Lastly, the complexity of the tumor microenvironment and its potential influence on FGL1 and IMPDH1 regulation necessitate additional investigation to fully comprehend the interplay between these molecules and the tumor immune response.

## Conclusion

In conclusion, our study demonstrates that radiation-induced upregulation of FGL1 promotes ESCC metastasis through IMPDH1. This research not only elucidates the molecular intricacies of radiation-induced ESCC metastasis but also unveils potential prognostic markers and therapeutic targets. While our findings hold substantial clinical promise, further investigation, including planned clinical trials, is imperative to validate these insights and advance them towards meaningful clinical application.

### Electronic supplementary material

Below is the link to the electronic supplementary material.

Supplementary Material 1

Supplementary Material 2

## Data Availability

The raw RNA-seq data has been deposited in the Gene Expression Omnibus (GEO) database under the accession number GSE264221. Other relevant data are provided within the manuscript or supplementary information files.

## Abbreviations

ESCC: Esophageal squamous cell carcinoma
FGL1: Fibrinogen-like protein 1
FOXO4: Forkhead box O4
IMPDH1: Inosine monophosphate dehydrogenase 1
FBS: Fetal bovine serum
shRNA: short hairpin RNA
qRT-PCR: quantitative real-time polymerase chain reaction
PBS: Phosphate-buffered saline
SEM: Standard error of the mean
NC: Negative control
OS: Overall survival
HR: Hazard ratio
CI: Confidence interval
