# Supplementary material for: Radiation-induced upregulation of FGL1 promotes esophageal squamous cell carcinoma metastasis via IMPDH1
Source: BMC Cancer. 2024 May 3;24:557. doi: 10.1186/s12885-024-12313-7 (PMC11067193; doi:10.1186/s12885-024-12313-7)
Supplement: Supplementary file 1 — Supplementary Material 1 [file 12885_2024_12313_MOESM1_ESM.pdf]

Table S1. Primer sequences used for qPCR.

Gene		Sequence(5'-3')
FGL1	forward primer	CCTTGTTACCACCGCTCTGA
	reverse primer	TGGGCTCTGGAGAGGTTTGA
IMPDH1	forward primer	CAGCAGGTGTGACGTTGAAAG
	reverse primer	AGCTCATCGCAATCATTGACG
FOXO4	forward primer	GGAATGCCTGGGGAAATCAG
	reverse primer	TTGTGGCGGATCGAGTTCTT
β-Actin	forward primer	CTCCATCCTGGCCTCGCTGT
	reverse primer	GCTGTCACCTTCACCGTTCC

Table S2. Sequences of shRNA.

Name	Sequence(5'-3')	Lentiviral vector
sh-IMPDH1#1	CCGGCCTGAAGAAGAACCGAGACTACTCGAG TAGTCTCGGTTCTTCTTCAGGTTTTTG	GV248
sh-IMPDH1#2	CCGGGTGACGTTGAAAGAGGCAAATCTCGAG ATTTGCCTCTTTCAACGTCACTTTTTG	GV248
sh-IMPDH1#3	CCGGCGGAAGGTCAAGAAGTTTGAACTCGAG TTCAAACTTCTTGACCTTCCG	GV248
sh-FOXO4#1	CCGGCCTGGAGTGTGACATGGATAACTCGAGTT ATCCATGTCACACTCCAGGTTTTTG	GV248
sh-FOXO4#2	CCGGCCAGCTTCAGTCAGCAGTTATCTCGAGAT AACTGCTGACTGAAGCTGGTTTTTG	GV248
sh-FOXO4#3	CCGGCGTCCACGAAGCAGTTCAAATCTCGAGAT TTGAACTGCTTCGTGGACGTTTTTG	GV248
sh-NC	TTCTCCGAACGTGTCACGT	GV248

Table S3. List of candidate binding sites for the promoter region of FGL1 predicted by JASPAR.

Matrix ID	Name	Score	Relative score	Sequence ID	Start	End	Strand	Predicted sequence
MA0848.1	FOXO4	11.037	0.966854746	NC_000008.11:c17912365-17910366	1166	1172	-	ATAAACA
MA0848.1	FOXO4	11.037	0.966854746	NC_000008.11:c17912365-17910366	1307	1313	-	ATAAACA
MA0849.1	FOXO6	10.993	0.961235516	NC_000008.11:c17912365-17910366	1166	1172	-	ATAAACA
MA0849.1	FOXO6	10.993	0.961235516	NC_000008.11:c17912365-17910366	1307	1313	-	ATAAACA
MA0480.1	Foxo1	10.7362	0.905607522	NC_000008.11:c17912365-17910366	1216	1226	-	AGCTGTTTTTA
MA0849.1	FOXO6	10.6653	0.955625989	NC_000008.11:c17912365-17910366	698	704	-	GTCAACA
MA0848.1	FOXO4	10.6357	0.958921266	NC_000008.11:c17912365-17910366	698	704	-	GTCAACA
MA0157.2	FOXO3	9.83891	0.90545055	NC_000008.11:c17912365-17910366	719	726	-	GTAAAAAA
MA0157.2	FOXO3	9.69352	0.902518451	NC_000008.11:c17912365-17910366	1474	1481	+	GGAAACAA
MA0476.1	FOS	9.58744	0.916807256	NC_000008.11:c17912365-17910366	206	216	+	TATTATTCATT
MA0848.1	FOXO4	8.95918	0.925776009	NC_000008.11:c17912365-17910366	218	224	-	ATCAACA
MA0848.1	FOXO4	8.95918	0.925776009	NC_000008.11:c17912365-17910366	1640	1646	-	ATCAACA
MA0848.1	FOXO4	8.5719	0.91811942	NC_000008.11:c17912365-17910366	71	77	+	ATAAATA
MA0848.1	FOXO4	8.5719	0.91811942	NC_000008.11:c17912365-17910366	1456	1462	+	ATAAATA
MA0849.1	FOXO6	8.40093	0.916861502	NC_000008.11:c17912365-17910366	218	224	-	ATCAACA
MA0849.1	FOXO6	8.40093	0.916861502	NC_000008.11:c17912365-17910366	1640	1646	-	ATCAACA
MA0849.1	FOXO6	8.23405	0.914004601	NC_000008.11:c17912365-17910366	1821	1827	+	GTACACA
MA0848.1	FOXO4	7.68615	0.90060836	NC_000008.11:c17912365-17910366	1821	1827	+	GTACACA
