# Supplementary figures and images for: Radiation-induced upregulation of FGL1 promotes esophageal squamous cell carcinoma metastasis via IMPDH1
Source: BMC Cancer. 2024 May 3;24:557. doi: 10.1186/s12885-024-12313-7 (PMC11067193; doi:10.1186/s12885-024-12313-7)

Figure 1B

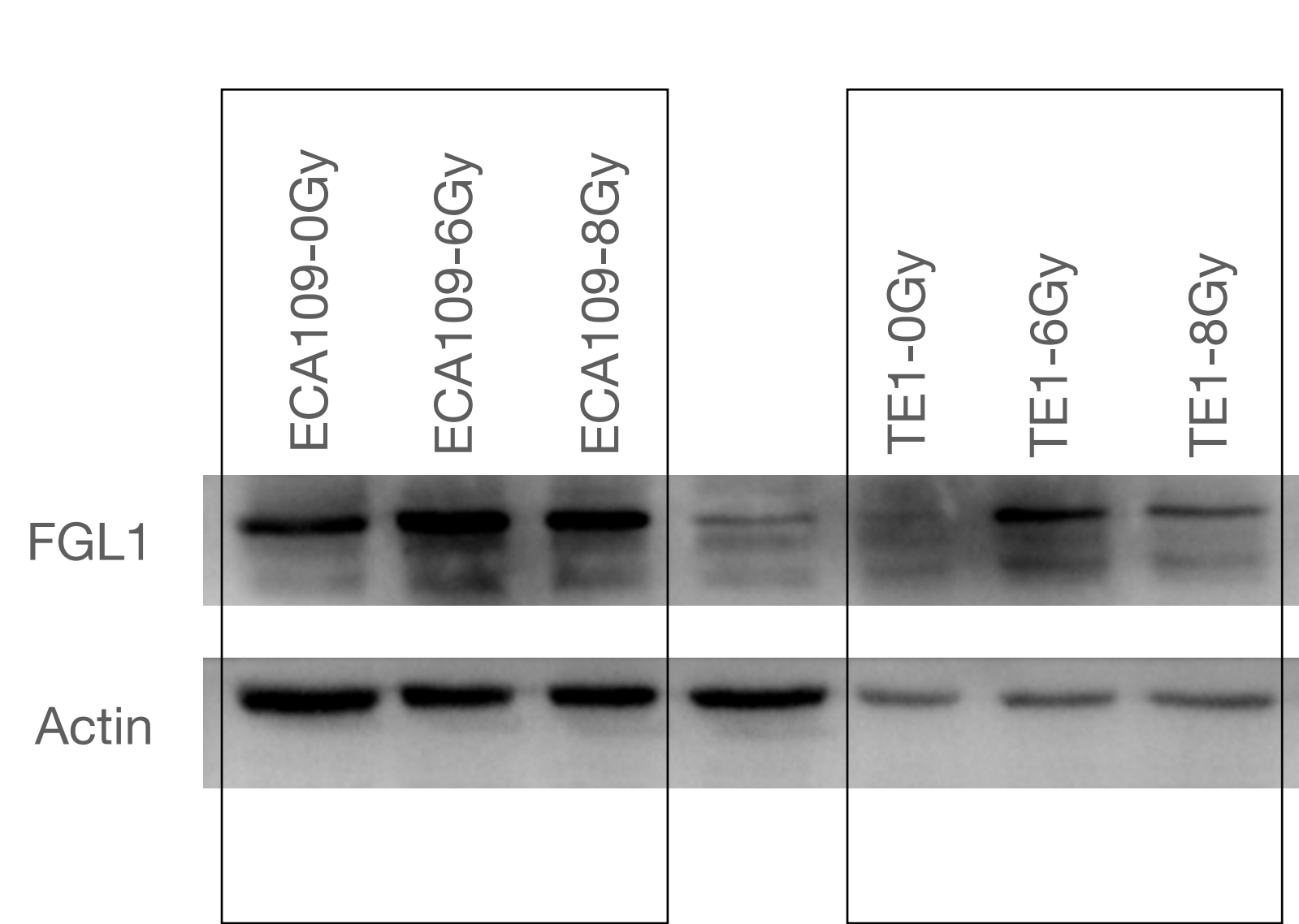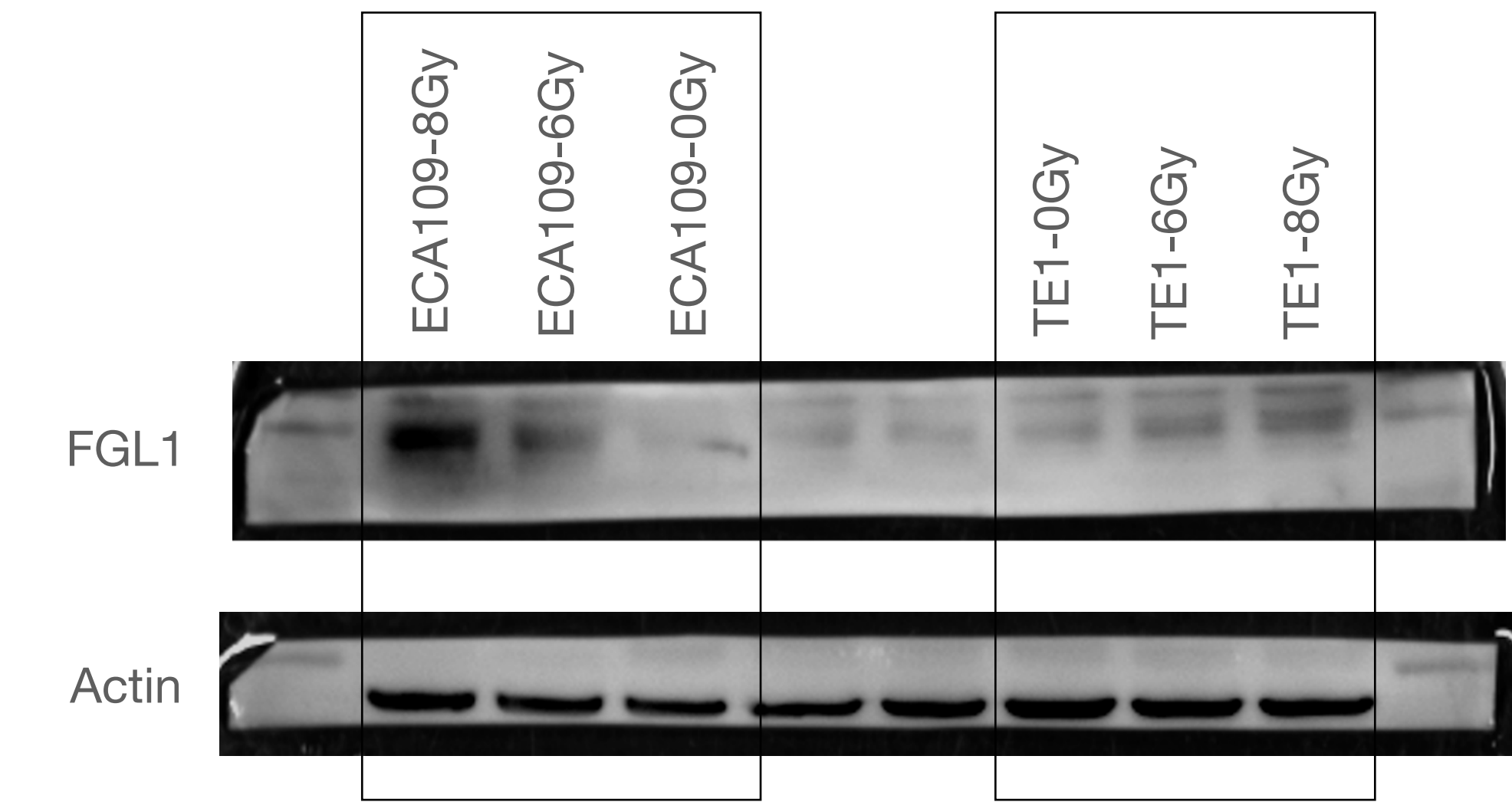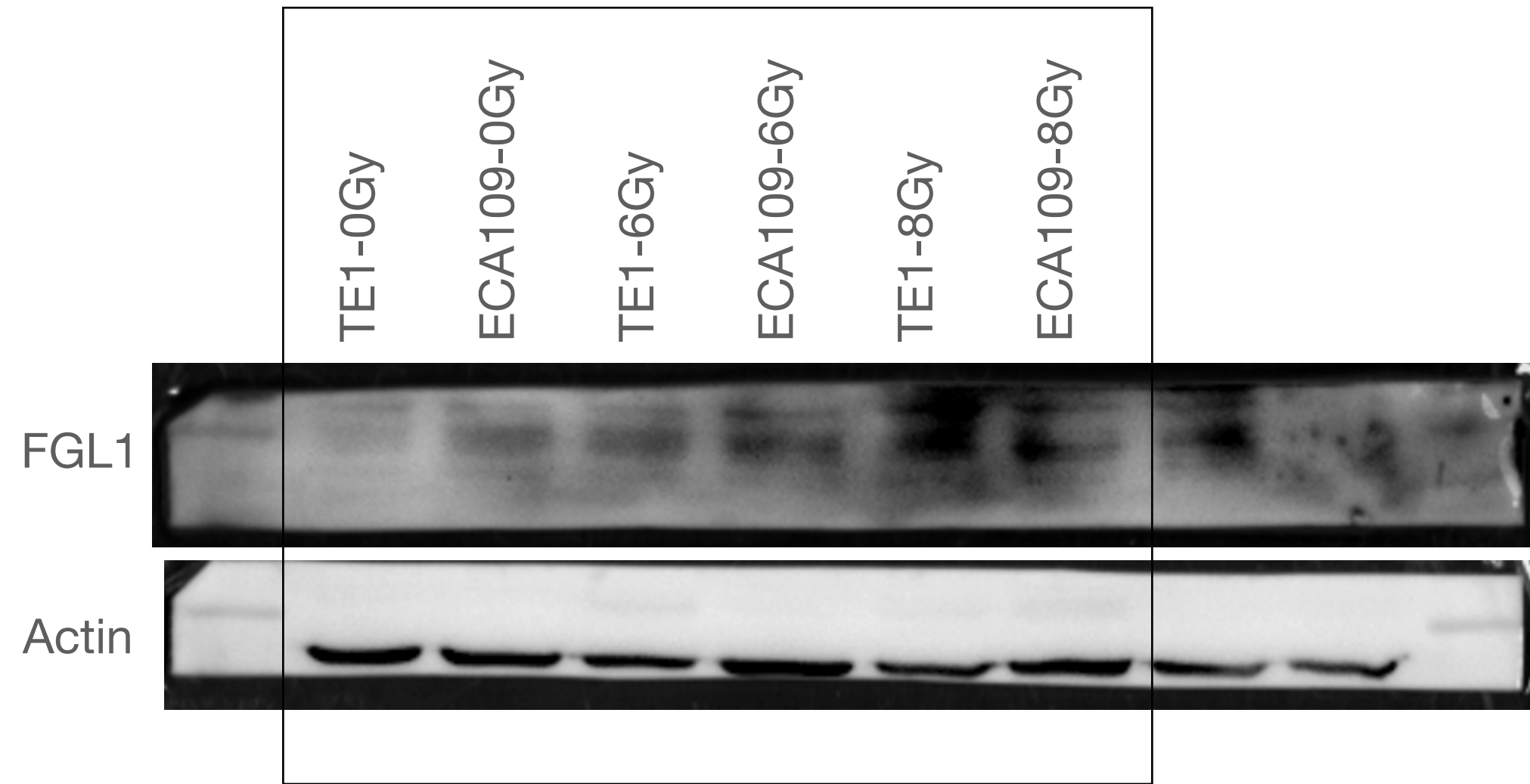

Figure 1H and 1K

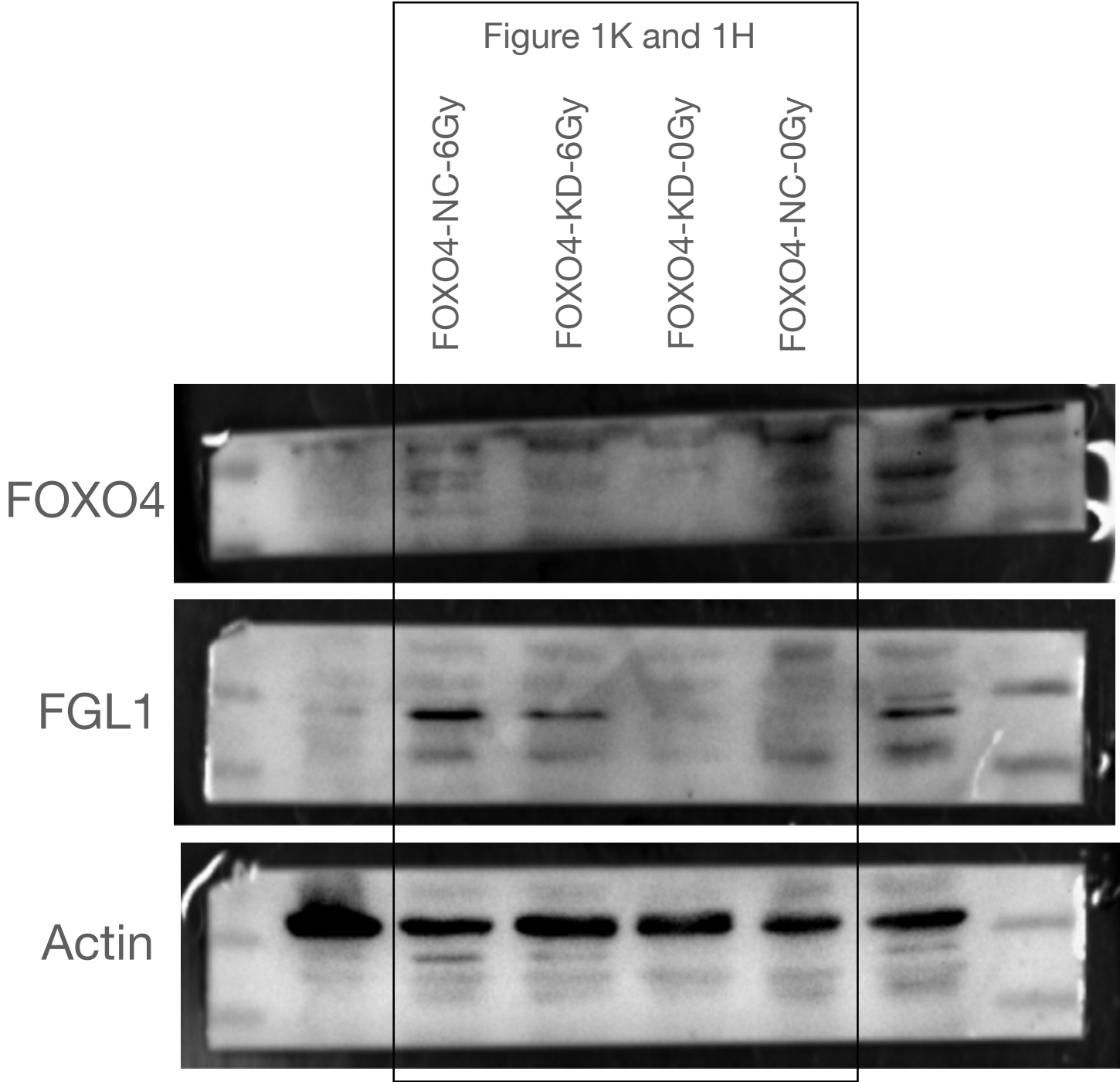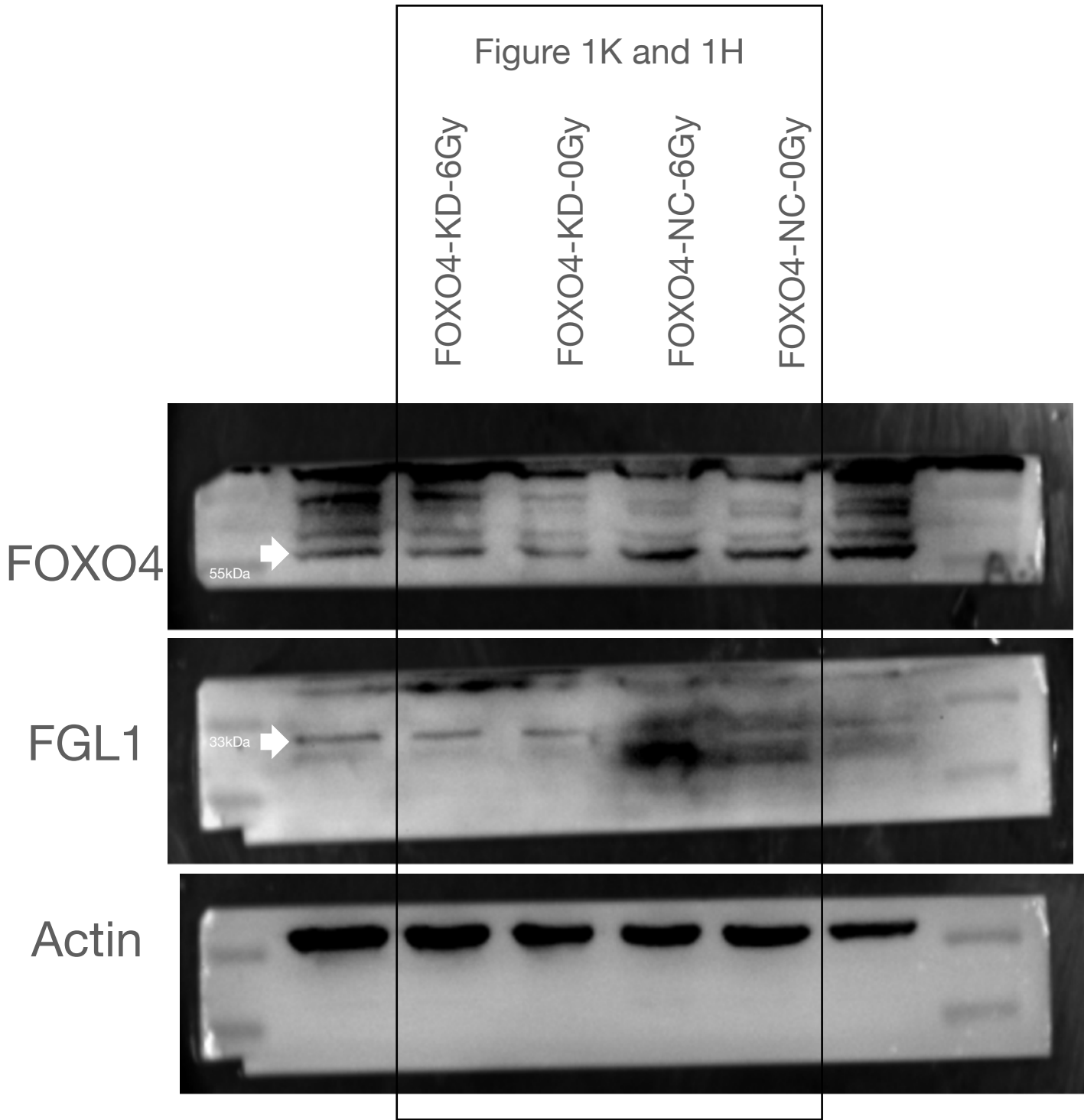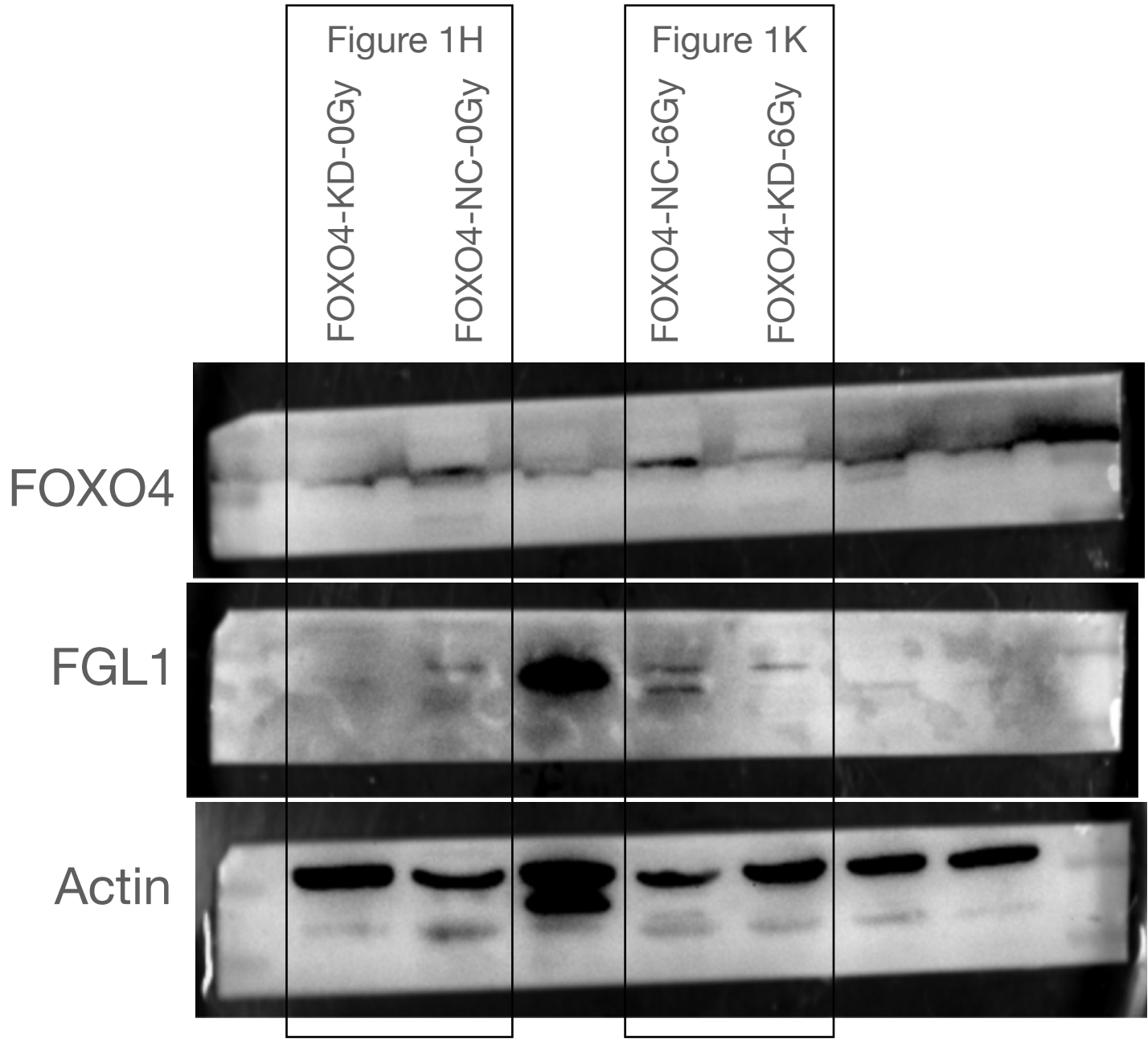

Figure 2A

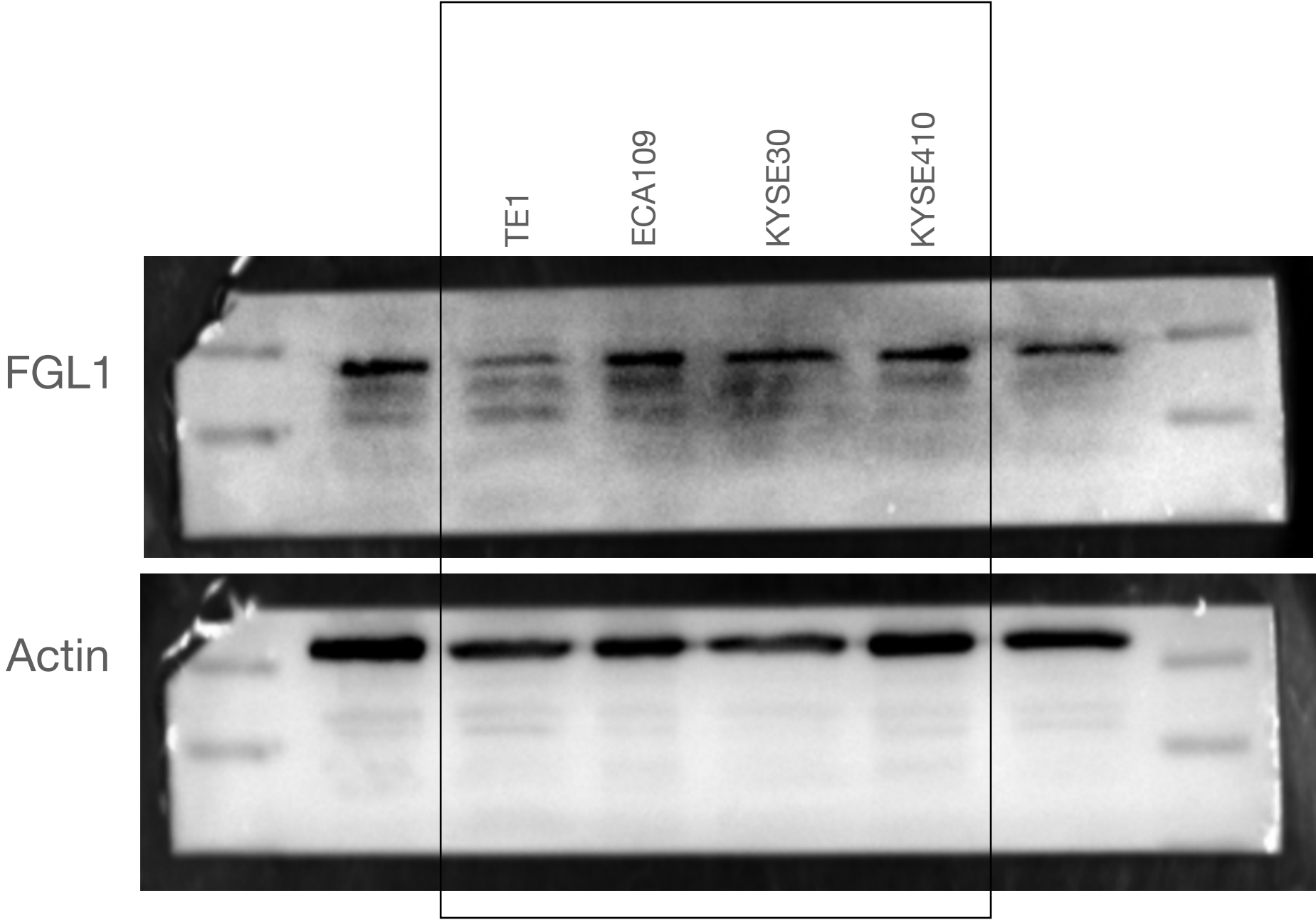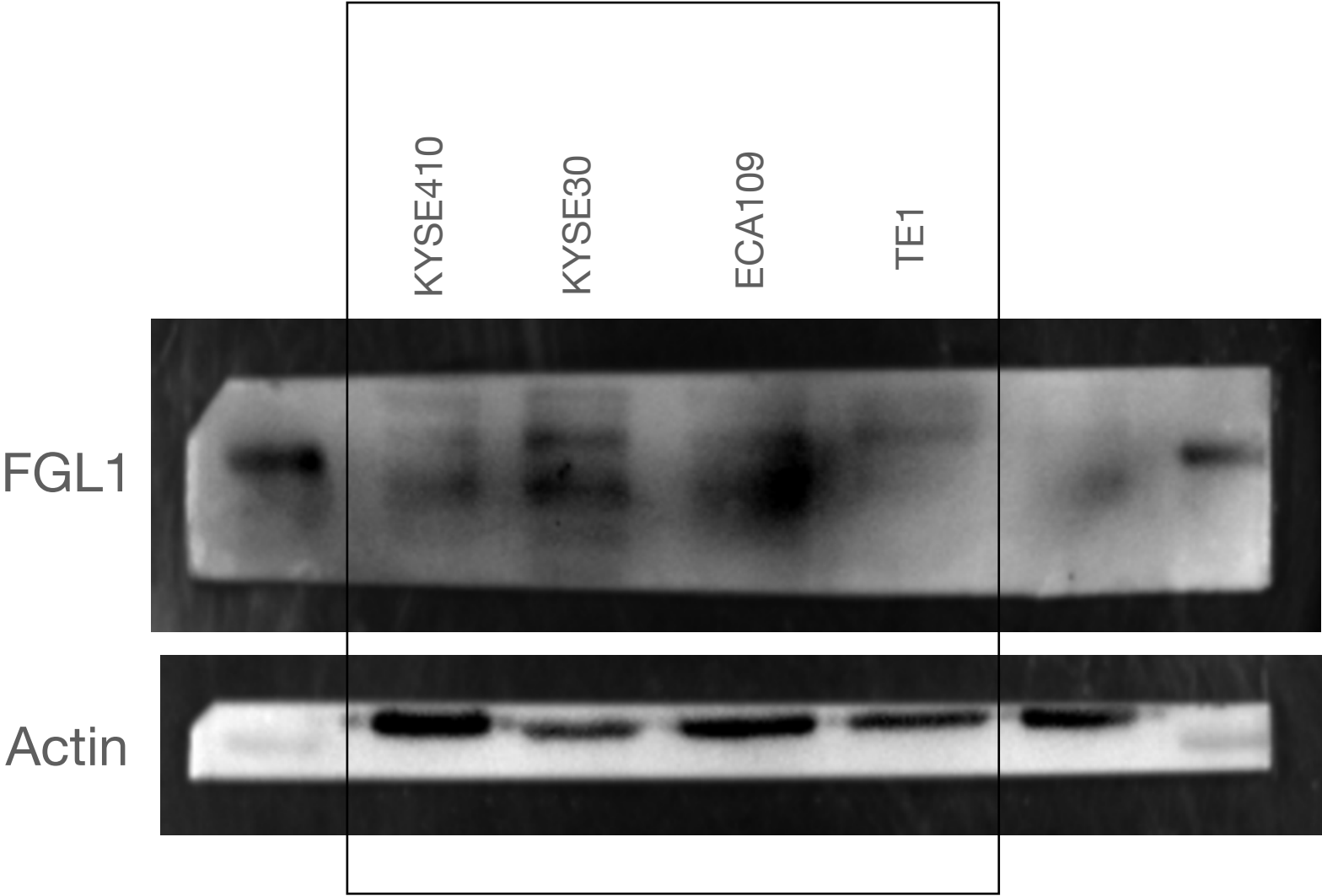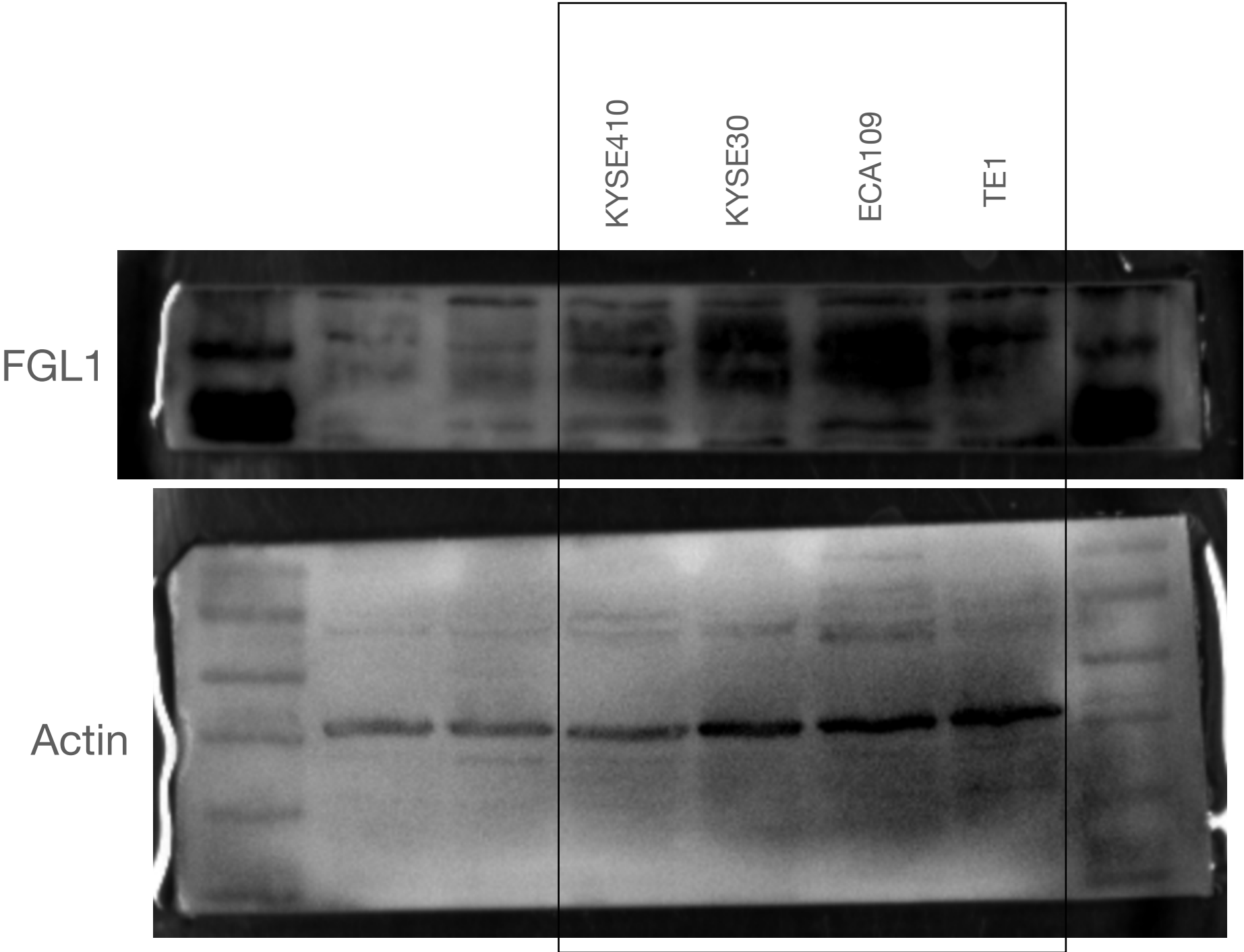

Figure 2D and Figure 3B

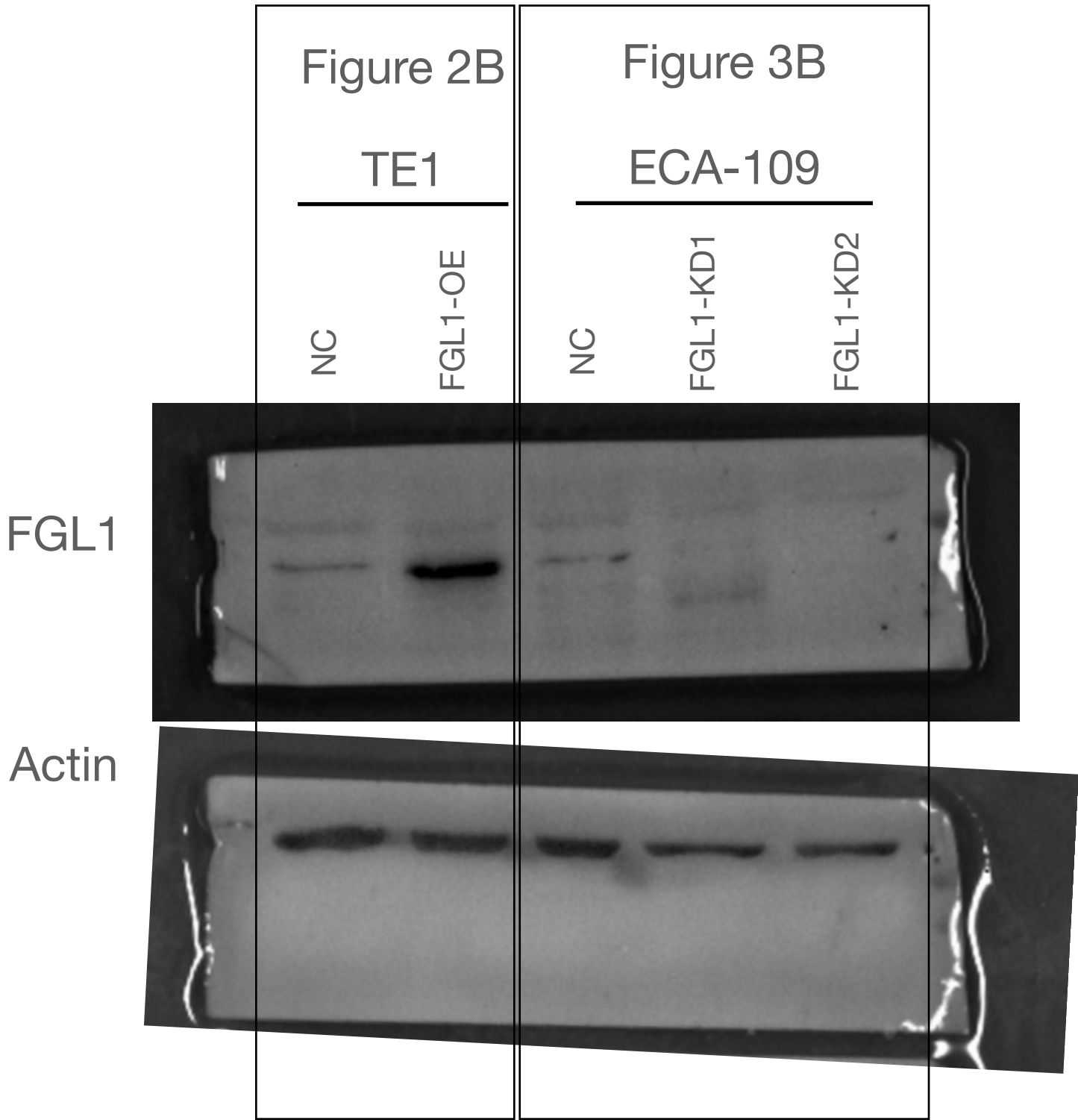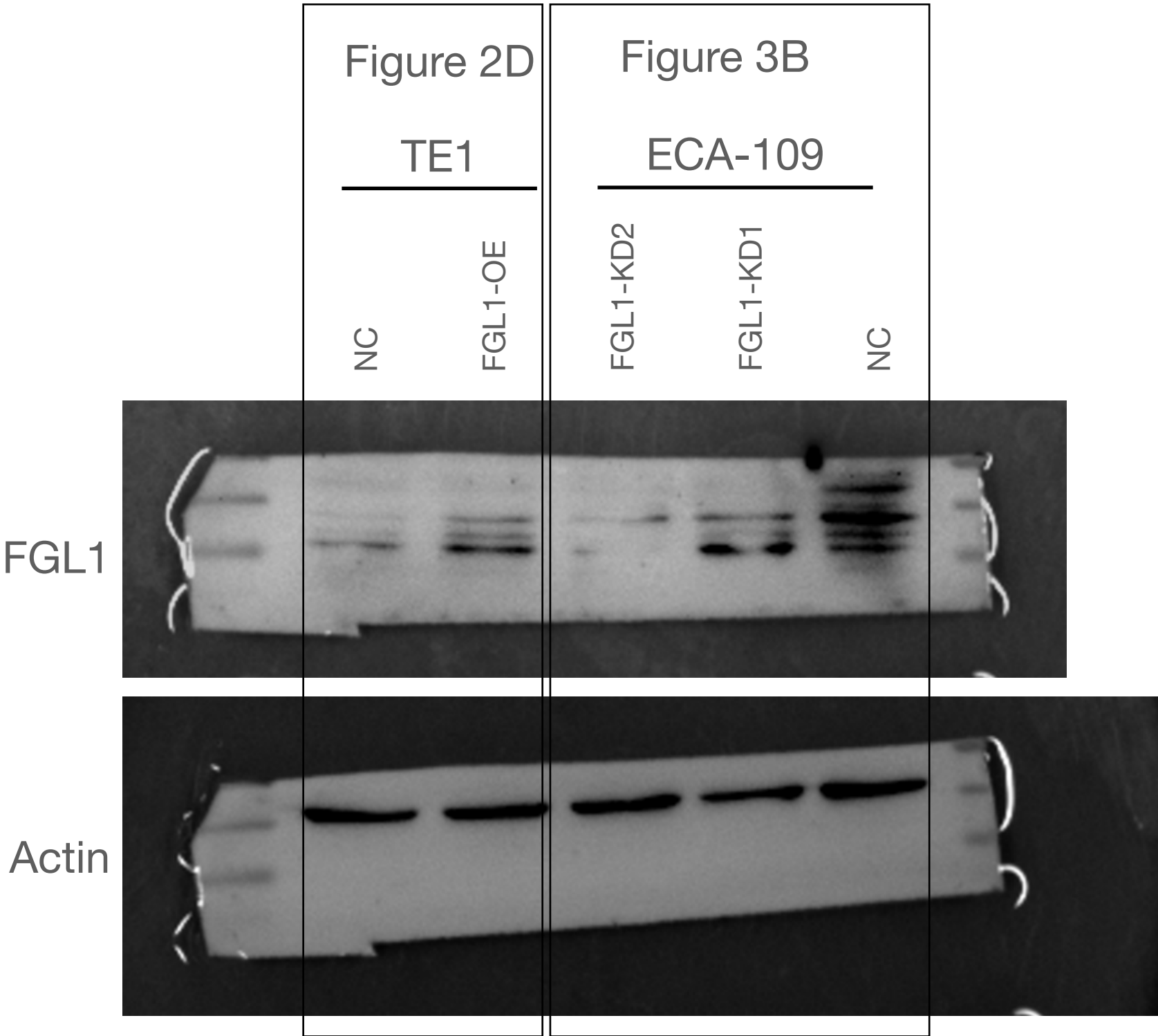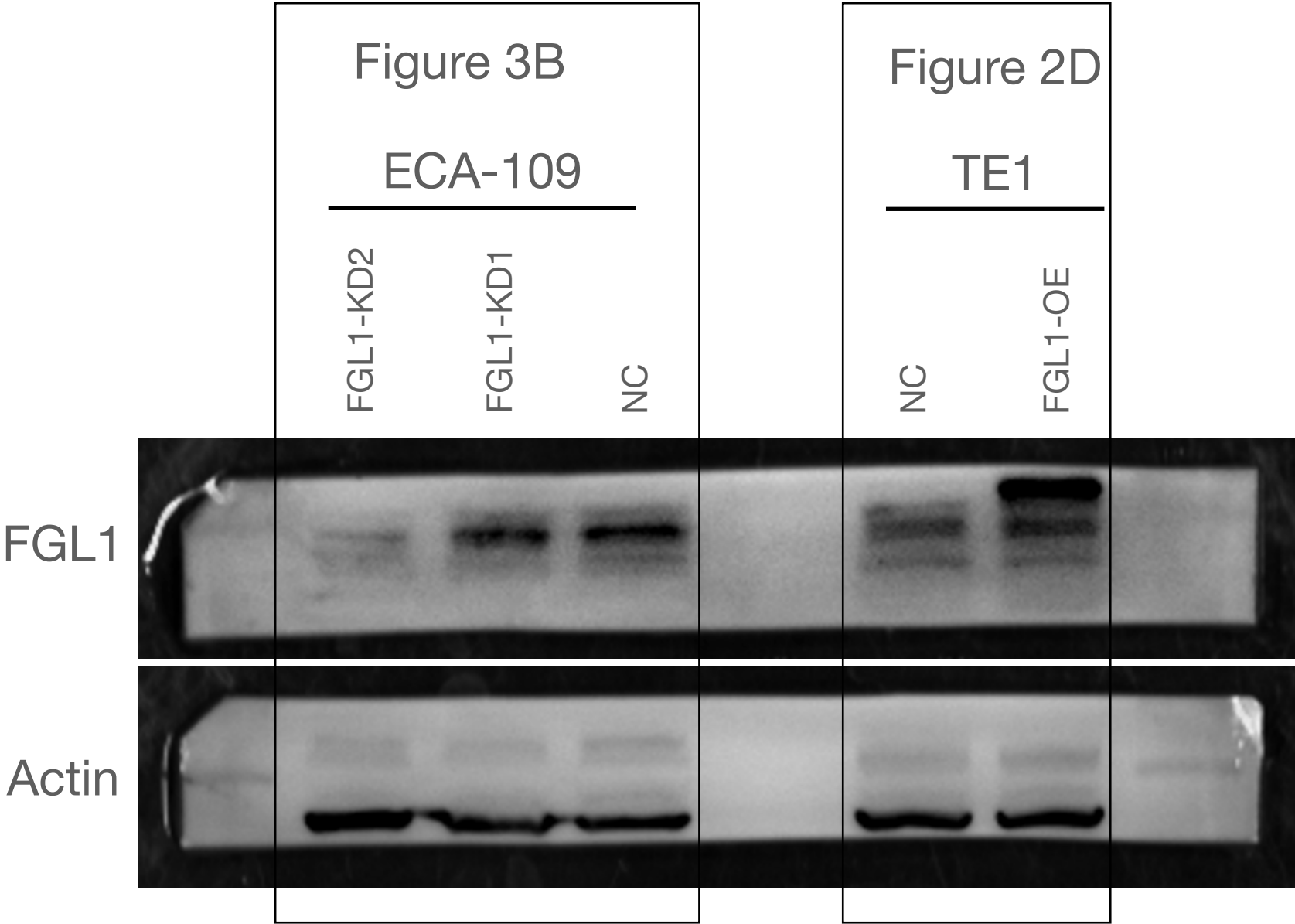

Figure 4D and Figure 5B

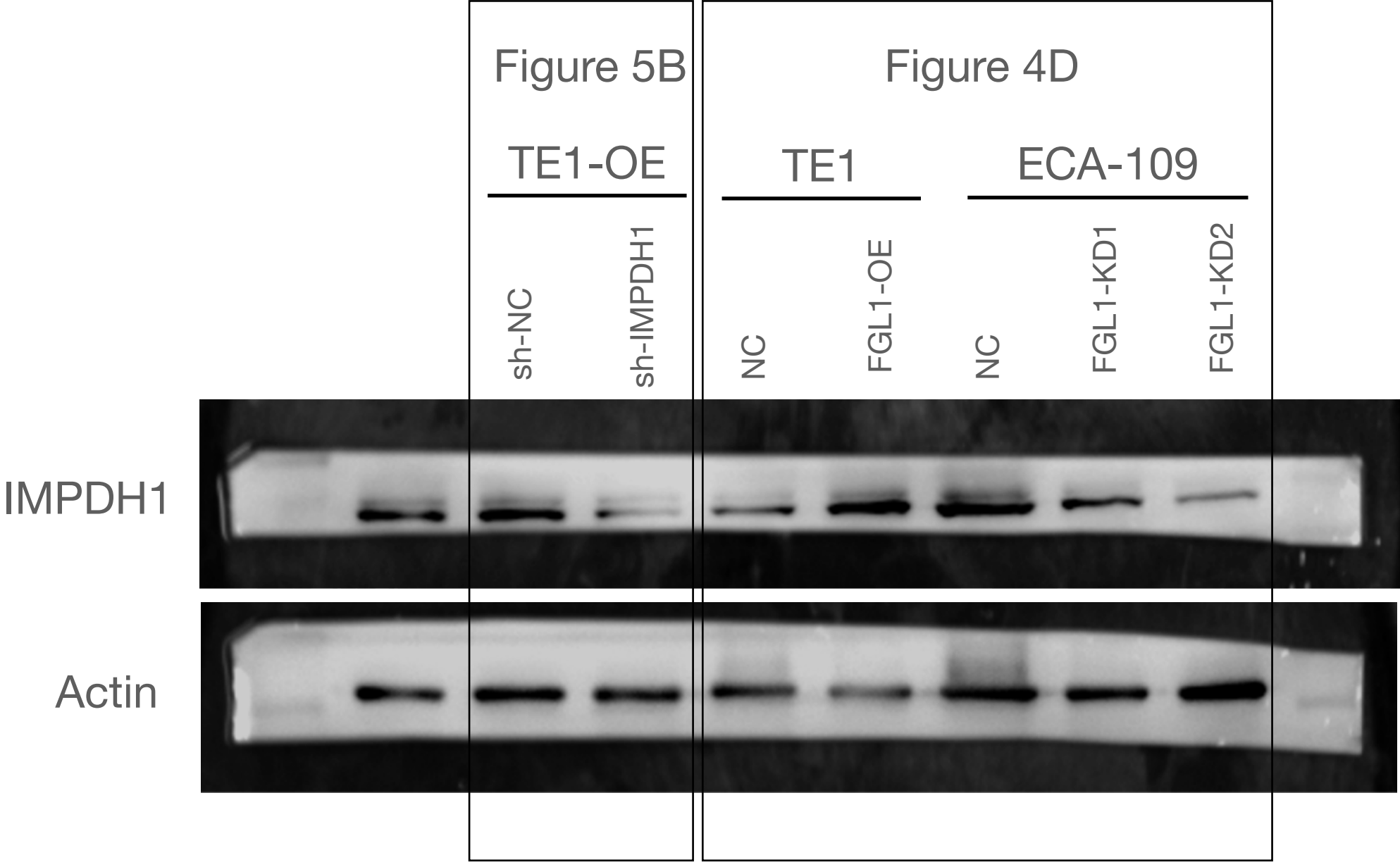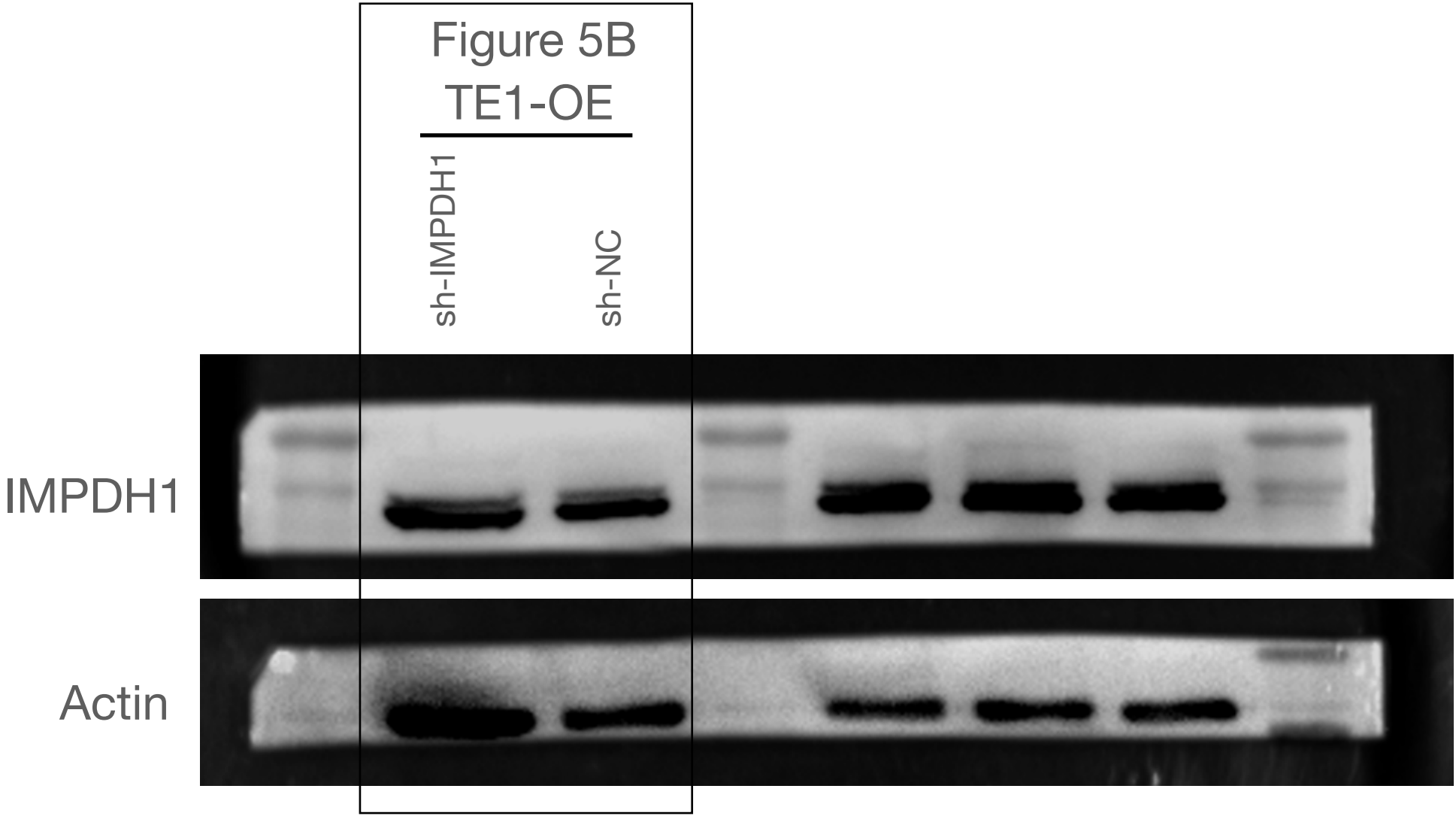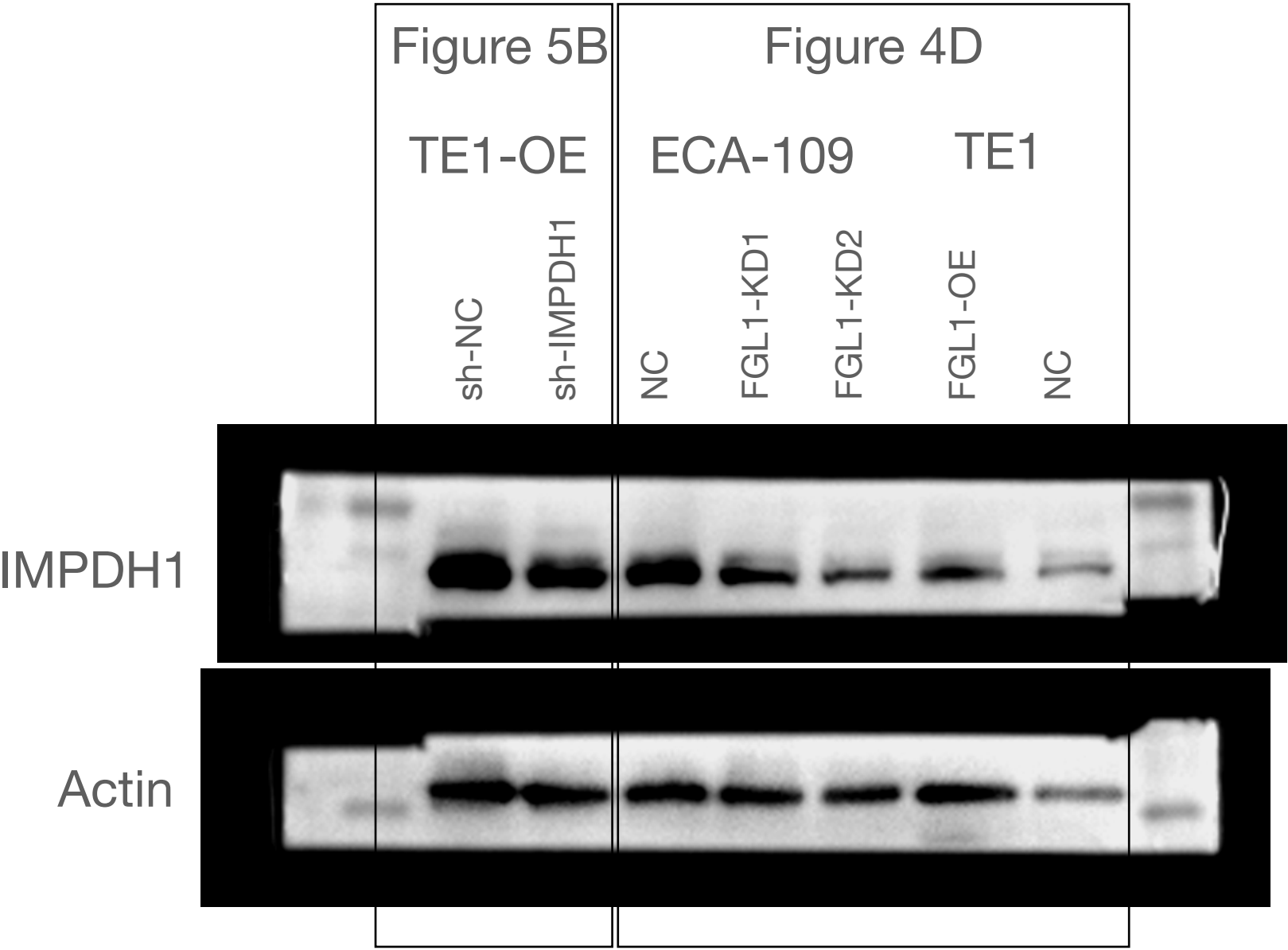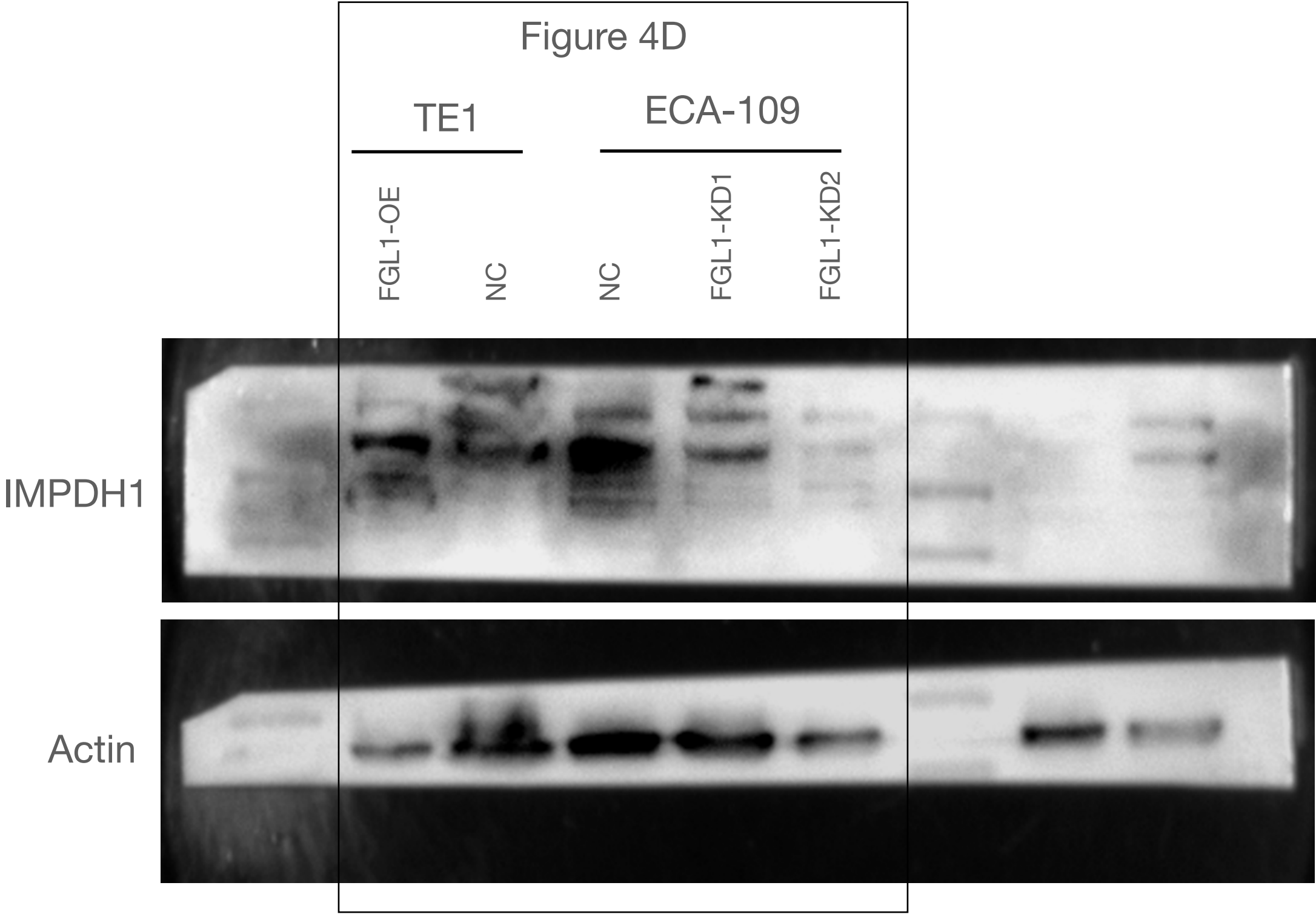

Figure 4G

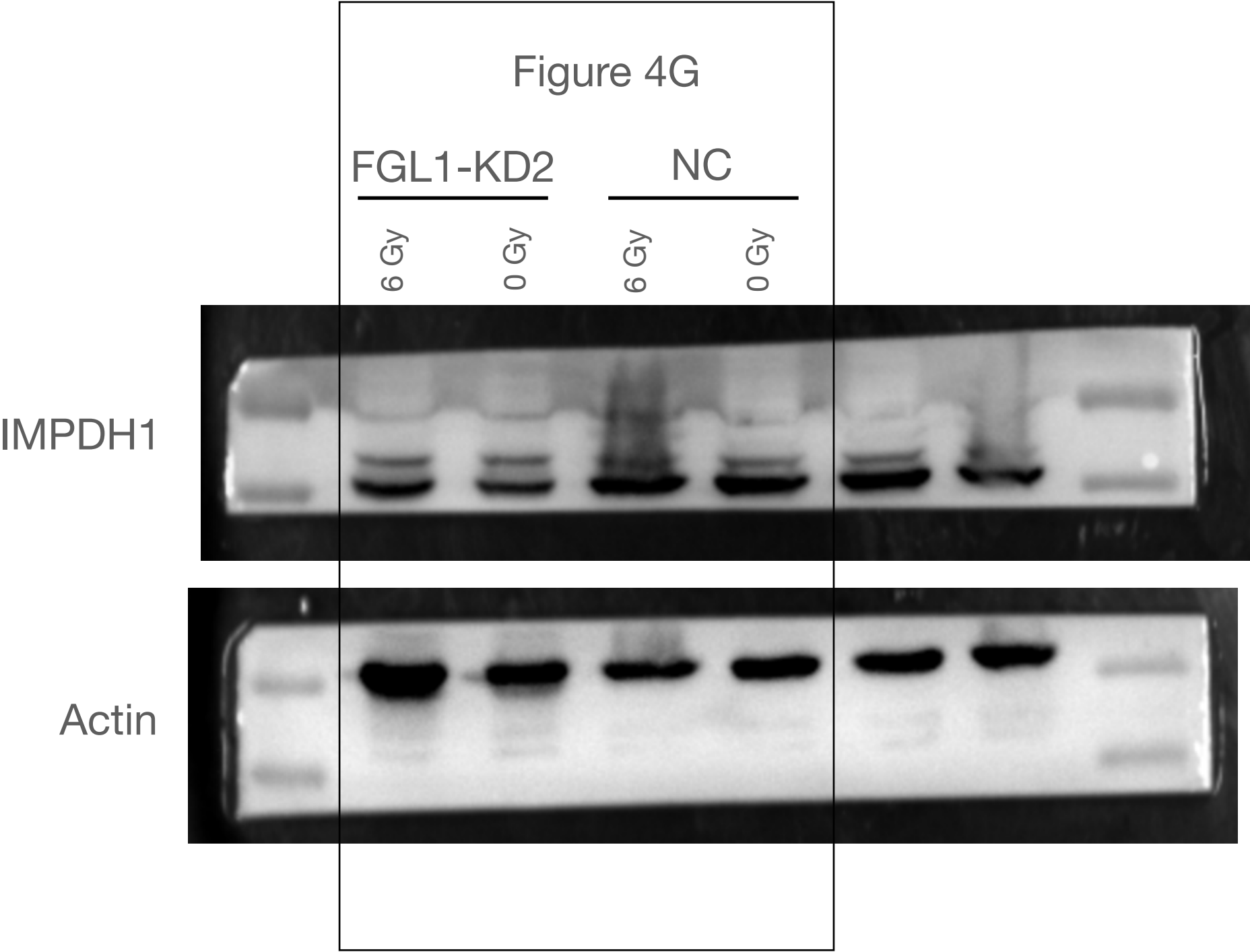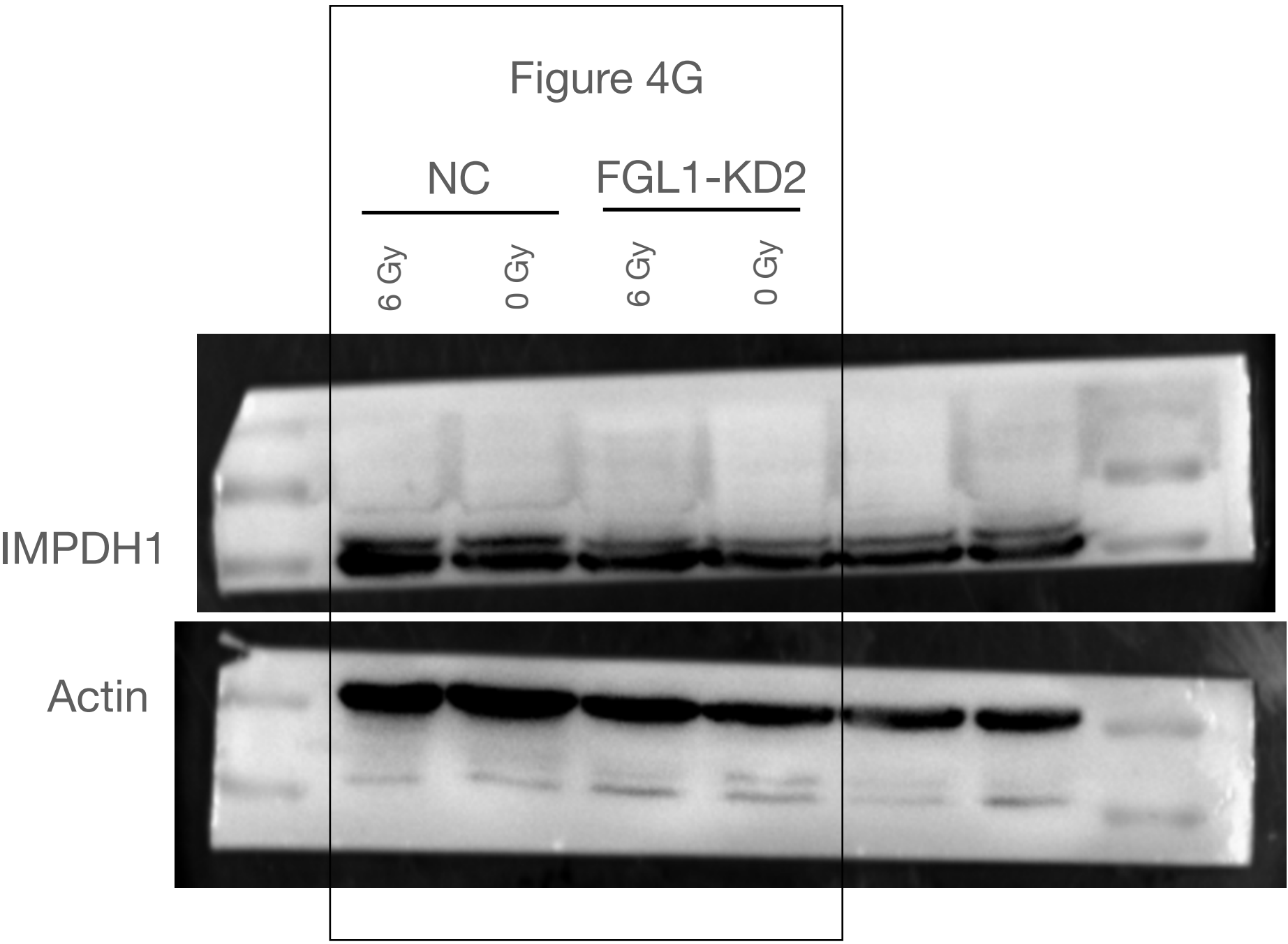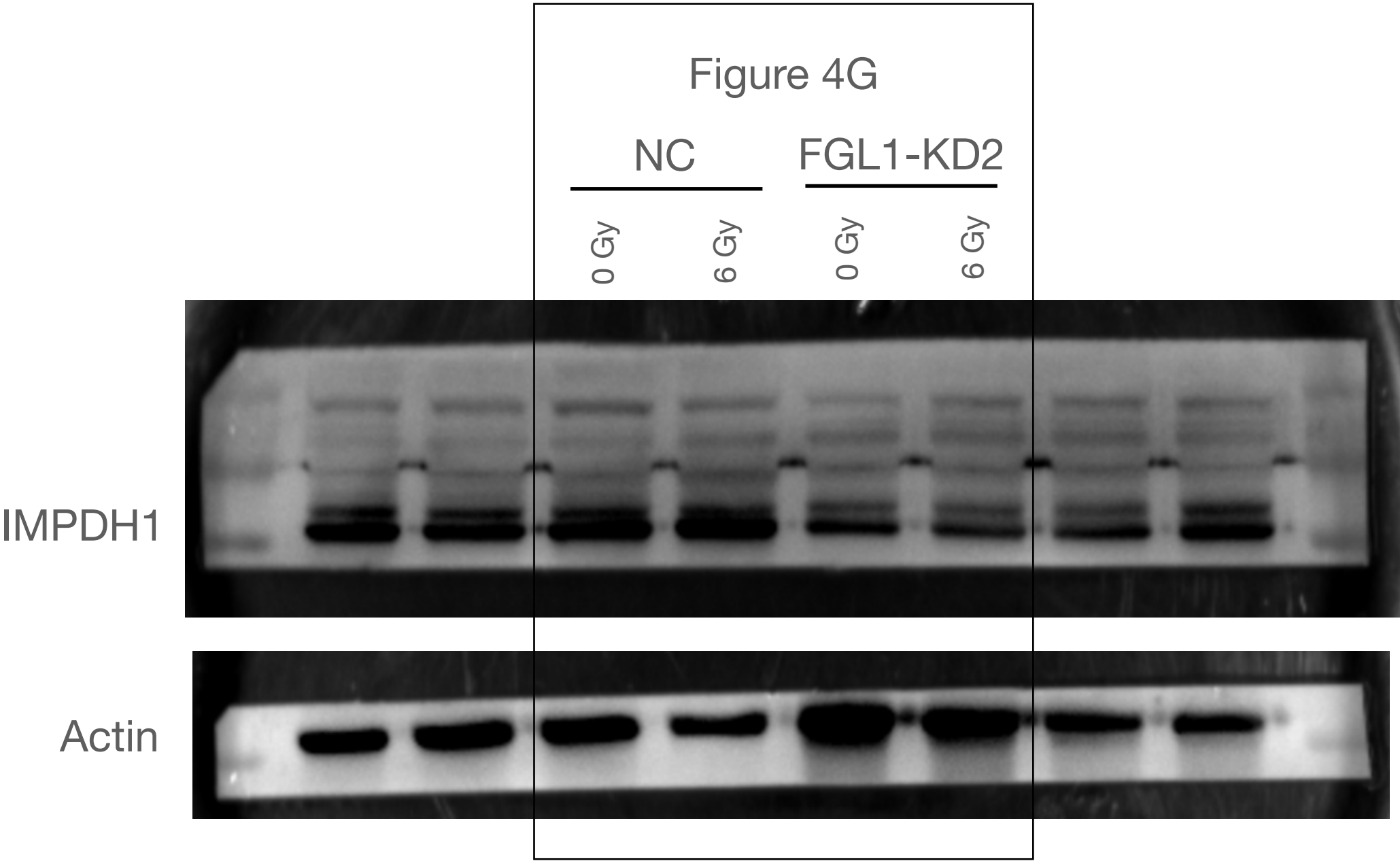

Supplement: Supplementary file 2 — Supplementary Material 2 [file 12885_2024_12313_MOESM2_ESM.pdf]
